# Climate-sensitive patterns of premature cardiovascular mortality in a subtropical city

**DOI:** 10.1371/journal.pone.0357804

**Published:** 2026-10-28

**Authors:** Noélia de Moraes Aguirre Carnasciali, Fábio Teodoro de Souza, Demian da Silveira Barcellos

**Affiliations:** 1 Curitiba Urban Planning and Research Institute (IPPUC), Curitiba, Brazil; 2 Graduate Program in Urban Management (PPGTU), Pontifical Catholic University of Paraná (PUCPR), Curitiba, Brazil; 3 Graduate Program in Smart and Sustainable Cities (PPGCIS), Pontifical Catholic University of Paraná (PUCPR), Curitiba, Brazil; 4 Research Centre for Economics and Corporate Sustainability (CEDON), KU Leuven, Brussels, Belgium; Universidade Federal de Santa Catarina, BRAZIL

## Abstract

This study aimed to investigate climate-sensitive temporal patterns associated with premature mortality due to cardiovascular diseases (CVD), ischemic heart disease (IHD), and acute myocardial infarction (AMI) in a large subtropical city in southern Brazil. Daily premature mortality records (ages 30–69) from 2010 to 2019 were analyzed together with meteorological data, including temperature, humidity, atmospheric pressure, precipitation, wind, and solar radiation, as well as temporally aggregated climatic indicators. An exploratory analytical framework combining multivariate analysis, clustering, and classification rule mining was applied to identify temporal patterns and combinations of climatic conditions associated with mortality outcomes. The approach was designed to support pattern discovery and hypothesis generation rather than causal inference.Linear associations between individual climatic variables and mortality were weak. However, exploratory analyses revealed distinct climate-sensitive temporal patterns. Premature IHD mortality was more frequently associated with colder temperatures, elevated long-term humidity, and specific combinations of atmospheric pressure and temperature. In contrast, AMI mortality showed associations with intermediate maximum temperatures, lower atmospheric pressure, and moderate levels of solar radiation. The classification models demonstrated moderate performance across outcomes, with the highest accuracy observed for AMI, indicating that complex temporal patterns may not be captured by linear analyses alone. The findings demonstrate that climate-sensitive temporal patterns in premature cardiovascular mortality can be identified using exploratory, data-driven approaches. Although causal relationships were not assessed, the results highlight the value of integrating climatic indicators and temporal aggregation into exploratory environmental health research and hypothesis generation. This framework is transparent, reproducible, and applicable to other urban settings, particularly in low- and middle-income countries facing increasing climate variability.

## Introduction

Cardiovascular diseases (CVD) result from a complex interaction between genetic susceptibility, behavioral factors, and environmental exposures. In recent years, increasing attention has been given to the role of the natural environment—including temperature, humidity, solar radiation, atmospheric conditions, and geographic characteristics—in shaping cardiovascular risk and mortality. Environmental factors may influence cardiovascular health through multiple physiological mechanisms, including thermoregulation, blood pressure, vascular function, inflammation, and metabolic responses, while also interacting with traditional risk factors such as aging, hypertension, diabetes, and lifestyle behaviors. Consequently, environmental conditions have been recognized as important components of the human exposome and should be considered alongside conventional cardiovascular risk factors when investigating disease occurrence and mortality patterns [1–7].

The relationship between ambient temperature and health is well known. As verified in ecological time series studies, the mortality-temperature relationship has a “U” shape [5]. Mortality rates are lowest on days when average temperatures range between 15° and 25°C, progressively increasing as ambient temperature becomes hotter or colder [6]. A study in China reported that mild cold temperatures contributed to premature deaths from CVD and overall mortality [7]. In Rio de Janeiro, a case-crossover study found that the effect of temperature on CVD mortality varied at the individual level (age, sex, race, education level) and the neighborhood level (income, IDE, energy consumption, and population growth) [8]. In São Paulo, a subtropical city with moderate climatic conditions, evidence showed that mortality associated with cold conditions is more significant than that associated with hot conditions [9].

More recent research has focused on how mortality behaves under extreme temperatures and urban heat island (UHI) conditions. Climate change has contributed to rising urban temperatures by increasing the frequency, intensity, and duration of heat events, while urbanization amplifies these effects through the urban heat island phenomenon. The replacement of natural vegetation with impervious surfaces, increased heat storage in buildings and pavements, and anthropogenic heat emissions reduce nighttime cooling and increase both average and minimum urban temperatures [10]. Several epidemiological studies have reported that both cold and hot temperatures are associated with all-cause mortality [7,11] and cardiovascular mortality [8,12].

In a metropolis in southern Brazil, the presence of heat islands was observed in 2019, with higher temperatures predominantly in the urban area, exceeding 29°C, and lower temperatures in regions with higher vegetation density, up to 10°C cooler [13].

Seasonal variations in the natural environment play an important role in regulating cardiovascular function and the incidence of CVD. Seasonal changes influence sun exposure, physical activity, dietary habits, and physiological responses that affect cardiovascular health [1,6]. Blood pressure and lipid profiles have been shown to vary seasonally, generally reaching higher levels during winter than summer, contributing to increased cardiovascular mortality during colder months [6,9]. Moreover, both cold and hot ambient temperatures have been consistently associated with acute cardiovascular events and cardiovascular mortality across different populations [5,7,8,12]. As climate change is projected to intensify temperature extremes and climate variability, these environmental exposures are expected to play an increasingly important role in the global burden of cardiovascular disease, particularly among vulnerable populations [4,10,12].

The relationship between sunlight exposure and cardiovascular health further illustrates the influence of environmental conditions on cardiovascular risk. Seasonal variation in cardiovascular mortality has suggested an important role for sunlight exposure, potentially mediated by biological mechanisms such as vitamin D synthesis, circadian rhythm regulation, and vascular function [1,6]. Recent studies have also reported that greater sunlight exposure and favorable atmospheric conditions may be associated with reduced cardiovascular mortality and improved cardiovascular health outcomes [14]. Together, these findings reinforce the importance of considering environmental exposures, including solar radiation, when investigating cardiovascular disease patterns.

The analytical framework adopted in this study builds upon previous research that has successfully applied data mining techniques to environmental and urban management problems. Previous studies have used association rule mining, classification rule mining, clustering, and predictive modeling to investigate rainfall-induced landslides [15], earthquake-triggered mass movements [16], wildfire occurrence [17], metropolitan land-use planning [18], respiratory morbidity associated with urban air pollution [19], and water quality monitoring optimization [20]. These studies demonstrated that data mining techniques are effective for identifying complex, non-linear relationships in large environmental datasets and for supporting decision-making through knowledge discovery. Data mining is part of the Knowledge Discovery in Databases (KDD) process, which aims to extract relevant and useful knowledge from datasets and validate the findings. Data mining is a central stage of the Knowledge Discovery in Databases (KDD) process, in which computational algorithms are used to identify hidden patterns, associations, and predictive relationships in large datasets that may not be detected using conventional statistical approaches [21,22].

In the clinical context of medicine, data mining is an expanding field and plays a fundamental role as a Computer-Aided Decision Support System (DSS) in supporting medical decisions [23]. Various methodologies have been tested to predict heart diseases by identifying individual risks based on clinical data. In the biomedical field, data mining predicts diseases by identifying redundant and interrelated symptoms and signs. In epidemiology, data mining is being developed to identify complex exposure patterns and health outcomes in the population. Decision trees and classification are used to analyze large epidemiological datasets and identify associations between environmental, behavioral, and biological exposures and disease occurrence [24]. These techniques allow for generating hypotheses about risk factors for certain diseases and aid in understanding the underlying mechanisms of the observed associations.

Previous studies have demonstrated the usefulness of data mining techniques for cardiovascular disease prediction, primarily using clinical and demographic variables. Systematic reviews have shown that decision trees, support vector machines, artificial neural networks, random forests, and other ensemble learning methods achieve promising predictive performance and support clinical decision-making by identifying individuals at increased cardiovascular risk [23,25]. For example, Chitra and Seenivasagam [23] highlighted the potential of classification algorithms for identifying high-risk patients, while Azmi et al. [25] concluded that although machine learning models improve predictive performance, they still rely predominantly on clinical and lifestyle variables, with limited incorporation of environmental exposures. In environmental epidemiology, classification and decision-tree approaches have also been successfully applied to identify complex relationships between environmental factors and health outcomes [24]. By integrating multiple environmental and temporal variables, data mining provides an opportunity to identify previously unrecognized patterns and generate hypotheses that may support environmental health surveillance and preventive strategies. Therefore, there is a need for analytical approaches capable of integrating environmental and temporal variables with conventional cardiovascular risk factors to better understand climate-sensitive cardiovascular outcomes.

This research aims to investigate the relationship between premature mortality from cardiovascular diseases (CVD), ischemic heart disease (IHD), and acute myocardial infarction (AMI) and the natural environment from a temporal perspective using a data mining approach. Although data mining has been widely applied in environmental sciences and disease prediction, its application to investigating associative patterns between environmental factors and premature cardiovascular mortality remains limited. We hypothesize that this analytical methodology enables a better understanding of the relationships between natural environmental variables and premature mortality from CVD, IHD, and AMI.

Curitiba, the capital of Paraná State in southern Brazil, has approximately 1.8 million inhabitants and is internationally recognized for its innovative urban planning and commitment to sustainability. Located on a plateau at approximately 934 m above sea level, the city has a subtropical climate that is milder than that of many other Brazilian cities. Covering approximately 43,000 hectares, Curitiba includes extensive green infrastructure, with an estimated 52 m^2^ of green area per inhabitant, well above the World Health Organization recommendation of 9 m^2^ per inhabitant [26].

Despite these characteristics and its well-established urban planning model based on land use, transportation, and the road system [27], the premature cardiovascular mortality rate in Curitiba remains comparable to those reported for other major Brazilian capitals. An exploratory study reported a cardiovascular mortality rate of 1.63 deaths per thousand inhabitants in Curitiba, similar to values observed in other large cities. This apparent paradox makes Curitiba an appropriate case study for investigating the relationship between environmental conditions and premature cardiovascular mortality.

## Methods

The selection of appropriate data sources, together with rigorous data preparation, is essential to ensure reliable analytical results in data mining studies. This research combined secondary health and environmental data and generated additional derived variables during preprocessing to support the identification of complex temporal patterns. Variables that are less frequently explored in studies of cardiovascular mortality, such as atmospheric pressure, wind, precipitation, and humidity, were intentionally included to broaden the environmental characterization. The procedures for data acquisition and preparation are described below.

### Data acquisition

The research encompasses two categories of data: health and environment. Regarding health data, premature mortality from cardiovascular diseases (CVD), ischemic heart disease (IHD), and acute myocardial infarction (AMI) were selected as the primary outcomes based on theoretical grounding and study objectives.

The Municipal Health Secretariat of Curitiba (Secretaria Municipal da Saúde – SMS) provided mortality data for Curitiba, stored in the Mortality Information System (SIM), from 01/01/2010 to 12/31/2019. Mortality was attributed to the Basic Cause of death recorded in the individual’s Death Certificate. The deaths were classified according to the International Statistical Classification of Diseases and Related Health Problems – 10th Revision (ICD-10). CVD mortality corresponds to deaths where the basic cause was due to diseases classified under codes I00-I99, IHD corresponds to deaths under codes I200-I259, and AMI corresponds to deaths under code I219, all of Chapter 9 of ICD-10.

The deceased’s age record was considered to determine if it was a premature death. For this research, premature death is considered to be one occurring in individuals aged 30–69 years. The following valid data were obtained for the study: 10,218 premature deaths from CVD, 3,826 premature deaths from IHD, and 2,599 premature deaths from AMI.

Environmental data for this research stage were requested from the Paraná Environmental Technology and Monitoring System (SIMEPAR) and collected from the Agritempo website (https://www.agritempo.gov.br). The data originate from a meteorological station in Curitiba, at the geographic coordinates of Latitude −25.44817 and Longitude −49.23033, with an Altitude of 935 meters. In cases where the same variable was available from both sources, the variable with fewer missing data points was used. This station was selected because it provides continuous, quality-controlled historical records covering the entire study period (2010–2019), ensuring temporal consistency across all meteorological variables analyzed. Although spatial variability in temperature exists within Curitiba due to urban heat island effects, the use of a single long-term reference station is consistent with the objective of characterizing city-scale temporal variations rather than neighborhood-scale microclimatic conditions.

Based on previous evidence demonstrating that multiple meteorological factors—including temperature, humidity, atmospheric pressure, precipitation, and wind—may influence cardiovascular outcomes [1,6,9,12], variables that are less frequently explored in studies of CVD mortality, such as atmospheric pressure, wind, rain, and humidity, were also included in the analysis.

The study’s time series covered the period from 01/01/2010 to 12/31/2019, totaling 3,651 days. In total, 18 variables were collected from the mentioned sources. Daily data were provided for the study period, the list of which is presented in Table 1.

**Table 1:** Details of the Natural Environment Domain Variables Collected for the Research.

NATURAL ENVIRONMENT DOMAIN				
Collected Variable		Unit	Variable name	Source
Sun light	Solar radiation	W/m2	solar_rad	SIMEPAR
Temperature	Average	ºC	avg_temp	SIMEPAR
	Minimum	ºC	min_temp	SIMEPAR
	Maximum	ºC	max_temp	SIMEPAR
Precipitation	Total	mm	mm_rain	SIMEPAR
	Drought	days	drought	Agritempo
Relative humidity	Average	%	avg_humidity	SIMEPAR
	Minimum	%	min_humidity	SIMEPAR
	Maximum	%	max_humidity	SIMEPAR
Atmospheric humidity	Minimum dew point	ºC	min_dew	Agritempo
	Maximum dew point	ºC	max_dew	Agritempo
Climate Humidity	Potential Evapotranspiration	mm	pot_evapotrans	Agritempo
Atmospheric pressure	Average	hPa	avg_pressure	SIMEPAR
	Minimum	hPa	min_pressure	SIMEPAR
	Maximum	hPa	max_pressure	Agritempo
Wind	Average speed	km/h	avg_wind	SIMEPAR
	Direction	°	direction	SIMEPAR
	Maximum Gust Speed	km/h	wind_gust_max	SIMEPAR

### Data preparation

The data preparation, or preprocessing stage, is crucial in transforming raw data into a format suitable for analysis. This step aims to manipulate and transform raw data to make its interpretation easier and more accurate [21]. The obtained databases were integrated using the date as the common key, resulting in a single daily database containing mortality records and their corresponding environmental variables. This integration ensured the consistency and integrity of the data used in subsequent modeling and mining.

In addition to the 18 environmental variables obtained from public agencies, 90 new variables were created. Most of these were derived from temporal lags. This approach was developed to better understand the relationship between exposure to specific environmental factors and the development of health outcomes over time. Multiple lag periods (2, 5, 7, 30, and 60 consecutive days) were evaluated to capture both short-term and cumulative environmental exposures. The maximum lag of 60 days was established as a methodological choice within the exploratory data mining framework to allow the evaluation of different temporal aggregation windows, rather than assuming a predefined biologically relevant exposure period. This strategy was adopted because previous studies have demonstrated that meteorological conditions may influence cardiovascular outcomes through different temporal and physiological pathways [1,6,9,12]. The new variables were therefore created to represent the average daily values over specific periods: 2, 5, 7, 30, and 60 consecutive days (see online supplement, S1 Table). For each day, lag variables were calculated using rolling windows over the preceding 2, 5, 7, 30, and 60 days. Arithmetic means were calculated for all continuous environmental variables, whereas cumulative sums were calculated for precipitation.

Additional derived variables were created according to the characteristics of each environmental indicator. The total daily precipitation in millimeters of rain allowed for calculating accumulated rain over 2, 5, 7, 30, and 60 consecutive days. Rainy days, a variable used to assess periods of precipitation, was a new variable derived from the data on droughts, i.e., days without rain. The daily thermal amplitude was calculated from the daily maximum and minimum temperature, the variation between the daily maximum and minimum temperature, along with their respective temporal lags over the mentioned intervals.

For modeling and data mining, other categorical variables were created to understand the seasonality of the phenomenon from the pre-existing data. For example, based on the dates of the individual’s death, it was determined in which season of the year the event occurred, whether it was a weekday or weekend, and the month. Wind direction, a numerical data, was categorized using nomenclature into North, South, East, and West. These derived variables were generated prior to the discretization stage and subsequently incorporated into the final analytical dataset.

The discretization stage was required for the Classification Based on Associations (CBA) algorithm employed in this study, as association rule mining operates on categorical attributes. Accordingly, continuous variables were transformed into discrete categories to enable rule generation and classification [21]. Discretization was performed using the statistical tercile division technique, dividing the data into three proportional groups based on the value distribution. The cut-off values were calculated independently for each continuous variable using its empirical distribution across the entire study period. The first and second terciles corresponded to the 33.3rd and 66.7th percentiles, respectively, resulting in three categories (low, intermediate, and high) containing approximately one-third of the observations for each variable.

### Data modeling

The data modeling phase in this study encompasses two main steps: multivariate analysis and classification rule mining.

The multivariate analysis includes a correlation matrix and two clustering techniques. The correlation matrix was constructed using only the original numerical meteorological variables to describe their linear relationships. In contrast, the clustering analyses were performed using the complete set of engineered numerical variables, including lagged and accumulated measures, because their objective was to explore similarity patterns across the full feature space generated during data preparation.This systematic approach to examining relationships among multiple numerical variables (both discrete and continuous) aims to explore associations and identify quantitative patterns [28]. The software Statistica (version 7.0, developed by StatSoft) and Python packages pandas, matplotlib.pyplot, and seaborn were used for these analyses.

A correlation matrix assesses the degree of correlation (linear relationship) between two or more variables, revealing hidden patterns and trends. This relationship is expressed by Pearson’s coefficient, ranging from −1 to +1 [29,30].

The correlation matrix was constructed using only the original meteorological variables and the outcome variables. Derived variables (lagged and accumulated measures) were intentionally excluded because they were generated in a subsequent feature engineering stage specifically for the classification models. Including these derived variables would have introduced multiple highly correlated representations of the same environmental measurements, reducing the interpretability of the descriptive correlation analysis. Therefore, their exclusion was not expected to bias the relationships presented among the original variables but rather preserved the exploratory purpose of this analysis.

Clustering is a multivariate analysis method that groups variables based on their similarities or dissimilarities. Two different clustering approaches were used: hierarchical cluster analysis with single-linkage and the k-means algorithm [31,32].

The Joining (tree clustering) method was implemented using the single-linkage algorithm and Euclidean distances. This hierarchical clustering method forms new groups by joining the most similar groups. A dendrogram was used to visualize these hierarchical relationships, and the resulting similarity patterns were interpreted through visual inspection for exploratory purposes, particularly concerning the relationship between environmental and dependent variables [21]. Because the objective of this analysis was exploratory rather than to determine an optimal cluster partition, formal cluster validation methods, such as the elbow method or silhouette analysis, were not applied.

K-means clustering is configured with a given parameter k, representing the desired number of clusters, k-means generates k clusters of n objects (the study variables). The goal is to maximize intra-cluster cohesion (similarity within each cluster) while minimizing inter-cluster separation (dissimilarity between clusters) [21]. The algorithm uses Euclidean distance and randomly partitions groups from the centroid, which can yield different results. The k value was selected to facilitate the exploratory interpretation of similarity patterns among variables rather than to optimize cluster validity.

Although several more recent machine learning algorithms are available, the objective of this study was not to maximize predictive performance but to identify transparent and interpretable relationships between environmental variables and premature cardiovascular mortality. Therefore, an associative classification approach was adopted because it generates explicit classification rules that facilitate knowledge discovery and support the interpretation of environmental patterns in a public health context.

Based on this objective, classification rule mining was performed using the CBA (Classification Based on Associations, version 2.0) software [33] to construct three classifier models. The models were designed to identify interpretable association patterns and generate hypotheses regarding the relationship between climatic variables and premature cardiovascular mortality, rather than to develop predictive models. Therefore, the CBA algorithm was selected because it generates transparent and easily interpretable IF–THEN classification rules. This software utilizes an integrated framework called associative classification. Classification rules are generated through association rule mining techniques, which is efficient as it reduces the number of rules obtained only by association methods and focuses the study (output or target category).

Initially, association rule mining identifies all database rules meeting minimum support (S%) and confidence (C%) constraints. These criteria are considered performance evaluators in data mining models, especially in association mining [21]. Setting a minimum support value statistically selects mutually exclusive rules covering a significant number of events. Subsequently, classification rule mining aims to find reduced rules to form an accurate classifier, seeking patterns and relationships among dataset variables to identify the target class or category [33].

### Classifier targets

The study’s classifier targets were defined as follows:

A) CVD Deaths: no deaths; days with 1–4 deaths; days with more than 4 deaths.
B) IHD Deaths: days with IHD deaths; days without IHD deaths.
C) AMI Deaths: days with AMI deaths; days without AMI deaths.

The thresholds adopted for the CVD outcome were defined according to the tercile distribution of the daily cardiovascular mortality counts, resulting in three categories that reflect the observed distribution of the data. Because daily IHD and AMI mortality counts were substantially lower, these outcomes were modeled as binary variables (presence/absence of deaths).

One of the main advantages of associative classification is that the resulting rules are directly interpretable, facilitating the identification of combinations of environmental conditions associated with each outcome class.

Classification rules are logical conditional models expressing a logical relationship between propositions, commonly symbolized as “A -> B” or verbally as “IF (A), THEN (B)” [34]. Support measures the frequency at which a particular association or pattern appears in the dataset, calculated as the percentage of times A and B simultaneously meet the rule condition relative to the total dataset records. Thus, a relevant pattern for the study’s 3651 days was a minimum frequency of 100 correct cases, with a minimum support (minisup) of 2.74% (100/3651). This support rate was used to evaluate performance across the three classifier models.

Confidence, the percentages A and B that meet the rule condition, is another performance measure in data mining [21]. Rules with less than 50% confidence were discarded, as they were not significant. Medium confidence (50%−75%) indicated moderate reliability, sufficient for discussion. High confidence (above 75%) showed strong reliability, suitable for decision-making and analysis [32].

In association rule mining, support and confidence are critical measures of interest. Criteria based on minimum values ensure rule relevance, though a minimum support criterion can eliminate many potentially interesting rules. Despite the importance of confidence, it should not overlook the support of the item set in the rule’s consequence.

Pruning parameters were empirically selected after preliminary testing to maximize classification performance for each outcome. The IHD classifier achieved better performance with pruning enabled, whereas the CVD and AMI classifiers showed higher accuracy without pruning.

“Pruning control” in data mining ensures model generalizability and avoids overfitting, leading to robust performance on unseen data [21]. This study established pruning control parameters: CVD and AMI Classifiers: “without pruning” and “use small rules.” IHD Classifier: “with pruning” and “use small rules.”

During model validation, the algorithm uses a set of association rules to evaluate the output variable and establishes classification rules. Results are presented in a confusion matrix, with correct classifications on the diagonal and incorrect classifications off-diagonal. Model accuracy, representing the correct classification rate, is calculated by summing correctly classified samples divided by the total number of samples used in validation [19]. Only models with accuracy above 65% were considered valid in this research.

## Results

### Multivariate analysis

For the multivariate analysis, 3651 rows of data were processed, corresponding to the days analyzed. The independent variables considered were the natural environmental variables. In the correlation analysis, only the numerical variables were evaluated. The selection of variables was restricted to the 18 collected variables, plus the temperature range variable, as the complete analysis did not yield different results from those of the dependent variables. The dependent variables considered in this study are the daily counts of premature mortality due to CVD, IHD, and AMI (count_cases_CVD, count_cases_IHD, and count_cases_AMI) during the study period from January 1, 2010, to December 31, 2019.

Fig 1 shows the correlation matrix of the variables in the form of a heatmap, where the value −1 is represented in blue (indicating a perfect negative linear correlation), and the value +1 is in red (indicating a perfect positive linear correlation). This analysis observed no significant linear association between the predictor (independent) and dependent variables (count_cases_CVD, count_cases_IHD, count_cases_AMI). It was observed that the dependent variables have a low correlation (value less than 0.3) with all other variables, except among themselves, where they exhibit moderate correlation. A high correlation (value greater than 0.7) was observed among some environmental (climatic) variables, consistent with the physical and atmospheric processes governing the climate.

**Fig 1:**
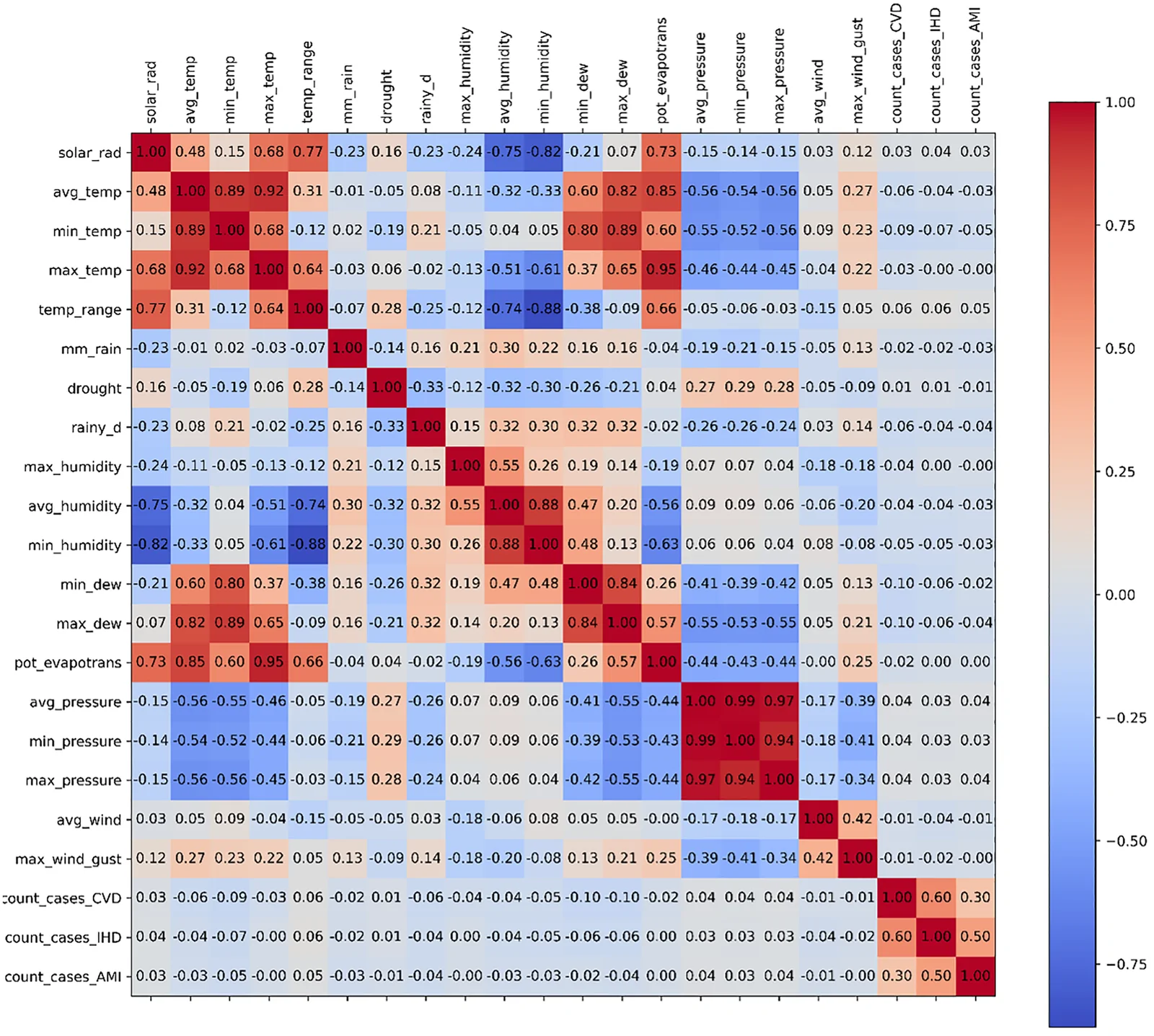
Correlation matrix of temporal (numeric) variables and daily counts of premature deaths due to CVD, IHD, and AMI in Curitiba from January 1, 2010, to December 31, 2019.

The hierarchical cluster analysis using the Joining method (tree clustering) depicted in Fig 2 provided insights into the relationships and similarities among variables, facilitating the understanding of clusters and their implications. Unlike the correlation analysis, which was restricted to the original numerical variables to describe their linear relationships, the clustering analysis included the complete set of 104 numerical independent variables, including the derived lagged and accumulated variables. These engineered variables were specifically created to capture delayed and cumulative environmental effects and to explore broader similarity patterns among the environmental conditions and premature mortality outcomes (count_cases_CVD, count_cases_IHD, and count_cases_AMI).

**Fig 2:**
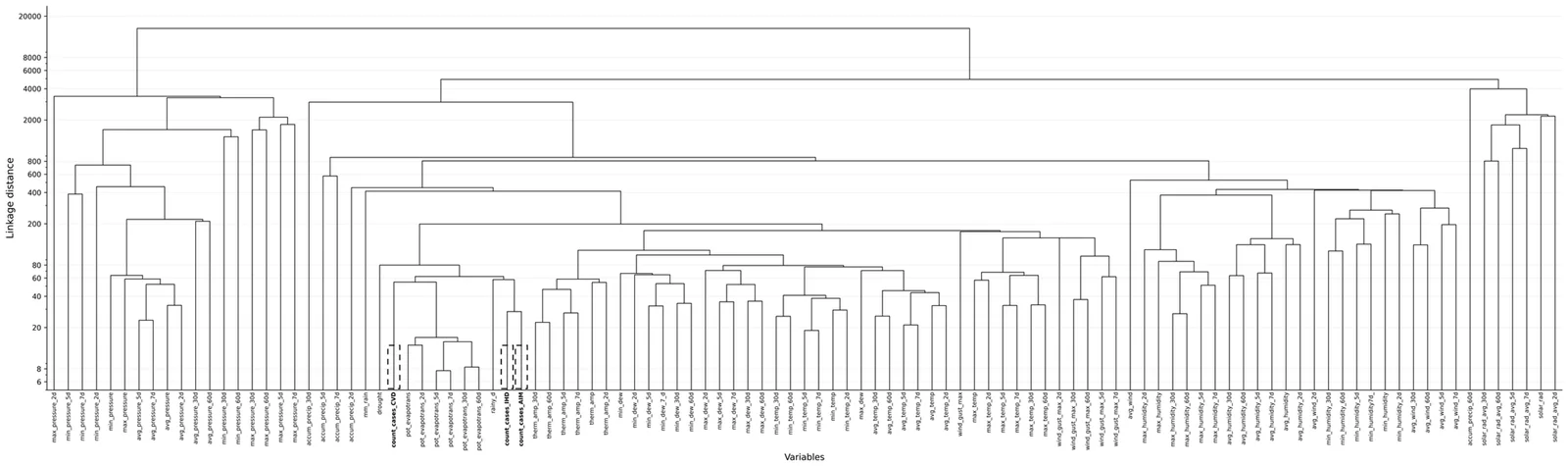
Dendrogram of the 107 variables (104 numerical environmental variables and three dependent variables representing premature mortality due to CVD, IHD, and AMI) in Curitiba from January 1, 2010, to December 31, 2019.

The presence of the dependent variables of premature mortality (count_cases_CVD, count_cases_IHD, count_cases_AMI) within the same cluster indicates a strong connection, suggesting similar patterns or mutual influence in specific contexts. Furthermore, the coexistence of mortality variables in this cluster with environmental variables such as dry days, rainy days, and daily evapotranspiration, including their temporal lags, reinforces the substantial similarity between these data sets.

The analysis of linkage distance (represented on the y-axis of the dendrogram) for the cluster containing the dependent variables highlights high intra-cluster similarity, indicating that variables in this cluster share common characteristics and behaviors compared to other clusters with a similar linkage distance value.

The central positioning of mortality variables on the x-axis suggests that those closer to the center are more similar in terms of mortality variables. Among these, it is important to highlight variables such as Rain, Rain Accumulated in 2 days, Temperature Range, Maximum relative humidity, Average Relative Humidity, Minimum dew point, Maximum dew point, and their respective temporal lags.

The number of group parameters in the k-means algorithm was determined based on the dendrogram analysis resulting from the cluster analysis. Visual inspection of the dendrogram revealed distinct groups of variables according to their hierarchical similarity. Because the objective of this analysis was exploratory rather than cluster optimization, formal cluster validation methods, such as the elbow method or silhouette analysis, were not applied.

Table 2 provides a comprehensive view of the distribution of variables across the identified clusters. Notably, the dependent variables, count_cases_CVD, count_cases_IHD, count_cases_AMI, were grouped in cluster 6, along with variables related to drought days, rainy days, daily evapotranspiration, and the average temporal lags derived from the latter. Interestingly, during the dendrogram analysis, these variables were previously identified in the same cluster as the mortality variables, emphasizing potential links or patterns of similarity among these data sets.

**Table 2:** Clustering of study variables using the k-means algorithm.

***Cluster 1*** ***(18 variables)***	max_pressure max_pressure_2d max_pressure_5d max_pressure_7d max_pressure_30d max_pressure_60d	avg_pressure avg _pressure_2d avg _pressure_5d avg _pressure_7d avg _pressure_30d avg_pressure_60d	min_pressure min _pressure_2d min _pressure_5d min _pressure_7d min _pressure_30d min _pressure_60d
***Cluster 2*** ***(7 variables)***	solar_rad solar_rad_avg_2d	solar_rad_avg_5d solar_rad_avg_7d solar_rad_avg_30d	solar_rad_avg_60d accum_precip_60d
***Cluster 3*** ***(25 variables)***	accum_precip_30d max_humidity max_humidity_2d max_humidity_5d max_humidity_7d max_humidity_30d max_humidity_60d avg_humidity	avg _humidity_2d avg _humidity_5d avg _humidity_7d avg _humidity_30d avg _humidity_60d min_humidity min _humidity_2d min _humidity_5d min _humidity_7d	min _humidity_30d min _humidity_60d avg_wind avg_wind_2d avg_wind_5d avg_wind_7d avg_wind_30d avg_wind_60d
***Cluster 4*** ***(14 variables)***	max_temp max_temp_2d max_temp_5d max_temp_7d max_temp_30d	max_temp_60d accum_precip_5d accum_precip_7d wind_gust_max wind_gust_max_2d	wind_gust_max_5d wind_gust_max_7d wind_gust_max_30d wind_gust_max_60d
***Cluster 5*** ***(32 variables)***	avg_temp avg_temp_2d avg_temp_5d avg_temp_7d avg_temp_30d avg_temp_60d min_temp min_temp_2d min_temp_5d min_temp_7d min_temp_30d	min_temp_60d temp_range temp_range_2d temp_range_5d temp_range_7d temp_range_30d temp_range_60d mm_rain accum_precip_2d min_dew min_ dew _2d	min_ dew _5d min_ dew _7d min_ dew _30d min_ dew _60d max_dew max_ dew _2d max_ dew _5d max_ dew _7d max_ dew _30d max_ dew _60d
***Cluster 6*** ***(11 variables)***	drought rainy_d pot_evapotrans pot_evapotrans_2d	pot_evapotrans_5d pot_evapotrans_7d pot_evapotrans_30d pot_evapotrans_60d	*count_cases_CVD* *count_cases_IHD* *count_cases_AMI*

### Classification model

More sophisticated statistical approaches were employed to identify possible non-linear or more complex associations and to understand more intricate relationships between variables. To this end, three classifier models were developed for the study days:

A) Model to classify total deaths due to CVD into no deaths, between 1 and 4 deaths, and above 4 deaths.
B) Model to classify the presence or absence of premature deaths due to IHD.
C) Model to classify the presence or absence of premature deaths due to AMI.

The CVD classifier model identified 2059 classifications for deaths between 1 and 4 per day; 383 for deaths above 4 per day; and 6 for days without deaths, totaling 2448 correct classifications. Thus, the model accuracy was estimated at 67.05%. The model generated 318 classification rules, none of which met the predefined minimum support (2.74%) and minimum confidence (50%) criteria for evaluation. Therefore, no robust classification rules were identified for CVD mortality. For completeness and to facilitate comparison with the IHD and AMI models, the three rules closest to meeting these thresholds are presented in Table 3. These rules are reported for descriptive purposes only and were not interpreted as robust classification patterns. This result indicates that, for this study, natural environmental factors did not directly act as a determining factor in the daily count of premature deaths due to CVD between 2010 and 2019 in Curitiba.

**Table 3:** Classification models generated by the CBA model. For CVD, the reported rules did not meet the predefined support and confidence thresholds and are presented solely for descriptive comparison with the IHD and AMI models.

Classification models for premature deaths due to CVD			
Number Rule	Rules	C (%)	S (%)
318	temp_range_60d_ > _11, AND, 1_ < _drought_ > _4, THEN, deaths_CVD_ > _4	41.33	3.39
290	solar_rad_ < _250, AND, solar_rad_5d_ > _350, THEN, 1_ < _deaths_CVD_ < _4	72.35	2.43
292	92_ < _max_humidity_30d_ < _97, AND, max_humidity_60d_ > _97, THEN, 1_ < _deaths_CVD_ < _4	71.90	2.38
**Classification models for premature deaths due to IHD**			
180	min _pressure_5d_ > _912, AND, 78_ < _avg_humidity_60d_ < _80, THEN, with_deaths_IHD	75.53	4.82
204	max_temp_2d_ < _22, AND, 78_ < _avg_humidity_60d_ < _80, THEN, with_deaths_IHD	70.95	4.08
181	max_dew_ < _15, AND, 78_ < _avg_humidity_60d_ < _80, THEN, with_deaths_IHD	75.46	3.36
193	pot_evapotrans_2d_ < _2, AND, 10_ < _temp_range_5d_ < _11, THEN, with_deaths_IHD	74.34	3.09
201	850_ < _max_pressure_30d_ < _915, AND, 3_ < _max_dew _7d_ < _14, THEN, with_deaths_IHD	73.68	3.06
168	min_dew_ < _10, AND, 10_ < _temp_range_5d_ < _11, THEN, with_deaths_IHD	77.03	2.84
**Classification models for premature deaths due to AMI**			
515	solar_rad_avg_2d_ < _250, AND, avg_pressure_ < _911, THEN, without_deaths_AMI	59.22	5.01
422	2_ < _pot_evapotrans_2d_ < _3, AND, avg_pressure_2d_ < _911, THEN, without_deaths_AMI	63.73	4.76
467	912_ < _avg_pressure_7d_ < _914, AND, max_dew_ > 18, THEN, with_deaths_AMI	61.95	3.12
513	min_humidity_60d_>58, AND, rainny_d=1, THEN, without_deaths_AMI	59.32	2.87
474	pot_evapotrans_2d_ > 3, AND, 22_ < _max_temp_2d_ < _26, THEN, with_deaths_AMI	61.62	2.90
482	avg_humidity_2d_ < _77, AND, solar_rad_avg_7d_ < _270, THEN, with_deaths_AMI	61.40	2.87
510	28_ < _avg_wind_30d_ < _30, AND, 10_ < _min_dew_ < _13, THEN, with_deaths_AMI	59.50	2.74

The IHD classification model recognized 2458 correct classifications, comprising 803 days without IHD deaths and 1655 days with deaths. Thus, the model accuracy was 67.32%. The model generated 223 classification rules, and six rules were selected (Table 3) based on rule performance evaluation (minimum support and high rule confidence). All the selected rules applied to days with premature IHD deaths. Out of the total 3651 study days, days with IHD deaths accounted for 64% of the study days.

Regarding the results obtained, it is important to emphasize that the model for IHD mortality identified specific rules for days with deaths, indicating its ability to identify interpretable patterns related to IHD. Additionally, this model shows the potential to provide new insights into the relationship between environmental conditions and IHD mortality. These findings suggest that the model can identify specific environmental conditions associated with days on which premature IHD deaths occurred.

Out of the six rules generated for IHD, three related a variable located in a dendrogram position distant from the IHD mortality variable—indicating dissimilarity concerning the independent variable—with a nearby variable. For the classification models of IHD, the input variables were grouped according to their proximity to the events. It was observed that variables such as min_pressure_5d, max_temp_2d, and max_pressure_30d showed a more distant relationship (“away”) with IHD cases. In contrast, variables such as max_dew, min_dew, avg_humidity_60d, avg_humidity_7d, max_dew_7d, avg_humidity_60d, temp_range_5d, and temp_range_5d were closer (“next”) to the events. Additionally, pot_evapotrans_2d stood out for being in the same cluster (“same cluster”) as the cases, suggesting a strong association with IHD occurrences. This shows the importance of using non-linear analyses to identify other relevant factors. The remaining rules considered variables close to or in the same cluster as the mortality variable, reinforcing the similarity identified in the dendrogram.

The AMI classification model recognized 2566 correct classifications, with 1272 days without AMI deaths and 1293 days with deaths. Thus, the model’s accuracy was determined to be 70.25%, the highest among the classifiers in this study. This indicates slightly superior performance in AMI case classification compared to CVD and IHD. The model generated 522 classification rules. It produced ten rules with minimum support within the study’s defined threshold (2.74%), the highest number of rules meeting the minimum support criterion. However, all these ten rules showed medium confidence, with none exceeding 65%, suggesting that the model may struggle to identify rules with high confidence for AMI case classification.

Nonetheless, recognizing this limitation, the methodological approach adopted in this study may contribute to future investigations evaluating the relationship between environmental conditions and premature cardiovascular mortality, particularly when complemented by additional datasets and external validation. Additionally, three of the ten generated rules were discarded due to a lack of logical relationships among conditions, leaving seven rules to be analyzed (Table 3). Among these seven selected rules, four corresponded to days with deaths and three to days without deaths.

The result obtained for AMI demonstrates that despite the confidence level of the rules not allowing accurate classification of deaths based on the studied environmental variables, further studies should be conducted incorporating these variables through other approaches. Notably, the AMI models on days with deaths generated rules with variables that multivariate analyses, particularly the dendrogram, had not identified as being similar to the death count.

Similar to the IHD model, 2 out of the four rules generated for days with AMI-related deaths involved an environmental variable distant from mortality in the dendrogram but close to or within the same cluster as mortality. For the classification models of AMI, it was found that solar_rad_avg_7d and max_temp_2d showed a more distant behavior (“away”) in relation to AMI events. On the other hand, avg_humidity_2d was closer (“next”), while pot_evapotrans_2d was in the same cluster (“same cluster”) as the cases analyzed, indicating a possible direct relationship with AMI records.

The variables that were more distant and, therefore, lacked similarity were: Average atmospheric pressure over 7 days, Maximum temperature over 2 days, Average solar radiation over 7 days. The variables with closer proximity were Maximum dew point and Average wind speed over 30 days. This finding suggests that the relationship between premature AMI mortality may involve complex associations not explained by traditional analyses.

## Discussion

Although the correlation matrix analysis revealed a lack of linear correlation among the studied variables, the cluster analysis uncovered relevant patterns of similarity and grouping. Independent variables grouped with dependent variables in clustering are typically suitable as inputs for classification models based on rule mining [19,34]. This highlights the importance of exploring alternative, non-linear analytical approaches to better understand the relationships between natural environmental variables and premature cardiovascular mortality. The combination of clustering and classification methods allowed for a richer interpretation of patterns not evident through linear analysis alone, reinforcing the need for multidimensional models in environmental epidemiology.

Several meteorological variables exhibited high pairwise correlations because they represented different summaries (minimum, average, and maximum values) of the same physical phenomenon rather than independent environmental exposures. These variables were intentionally retained because each descriptor may capture distinct temporal characteristics relevant to cardiovascular outcomes, particularly after temporal aggregation and lag generation. Although correlated variables may influence distance-based exploratory methods and generate overlapping candidate rules in associative classification, the clustering analyses were used exclusively for exploratory pattern identification, whereas the CBA algorithm applies minimum support, confidence thresholds, rule ranking, and pruning procedures to reduce redundancy in the final classifier. Therefore, the retained variables were considered complementary descriptors of environmental conditions rather than independent predictors. Likewise, the hierarchical clustering was interpreted through visual inspection of the dendrogram, as its purpose was to explore similarity patterns rather than determine an optimal cluster partition. Consequently, formal cluster validation methods, such as the elbow method or silhouette analysis, were not applied. Future studies may incorporate these approaches to quantitatively assess cluster separation and stability.

Cluster 2 exhibited greater heterogeneity than the remaining clusters, grouping accumulated precipitation over 60 days with solar radiation variables despite representing different meteorological processes. This outcome reflects the unsupervised nature of k-means clustering, which groups variables according to statistical similarity across the dataset rather than their physical interpretation. Therefore, this clustering result should be interpreted as a statistical association rather than evidence of a direct environmental relationship. Furthermore, accumulated precipitation over 60 days did not emerge as one of the most influential variables in the final classification models, suggesting that its contribution to predicting premature cardiovascular mortality was limited.

The analytical models produced several significant classification rules, particularly for ischemic heart disease (IHD) and acute myocardial infarction (AMI). For IHD, patterns showed that average humidity over 60 days between 78–80%, when combined with specific atmospheric and thermal conditions—such as minimum pressure above 912 hPa (confidence: 75.53%, support: 4.82%), maximum temperature below 22 °C (confidence: 70.95%, support: 4.08%), or maximum dew point below 15 °C (confidence: 75.46%, support: 3.36%)—were predictive of increased premature mortality. Other rules associated IHD deaths with narrow temperature ranges and pressure-dew point combinations, pointing to cumulative and interacting environmental factors. For AMI, relevant patterns emerged under intermediate maximum temperature ranges (22–26 °C), low atmospheric pressure, and moderate solar radiation, reaching up to 70.25% classification accuracy. These insights emphasize the cumulative impact of environmental exposure on cardiovascular risk and align with previous research highlighting U-shaped associations between temperature and cardiovascular events [5,6,35].

The flowchart (Fig 3) illustrates the theoretical framework, previous findings, key results, and practical implications of this study, which explores the relationship between natural environmental conditions and premature cardiovascular mortality using data mining techniques. Based on established theories linking environmental exposures—such as ambient temperature, humidity, and solar radiation—to cardiovascular outcomes, the study applied multivariate and classification analyses to uncover non-linear patterns over a 10-year period in a major Brazilian city. While linear correlations were weak (r < 0.3), cluster and rule-based models revealed specific associations, particularly for IHD and AMI. These findings support the inclusion of environmental lag variables in public health surveillance and demonstrate a cost-effective and replicable methodology for guiding climate-sensitive health interventions and policy design.

**Fig 3:**
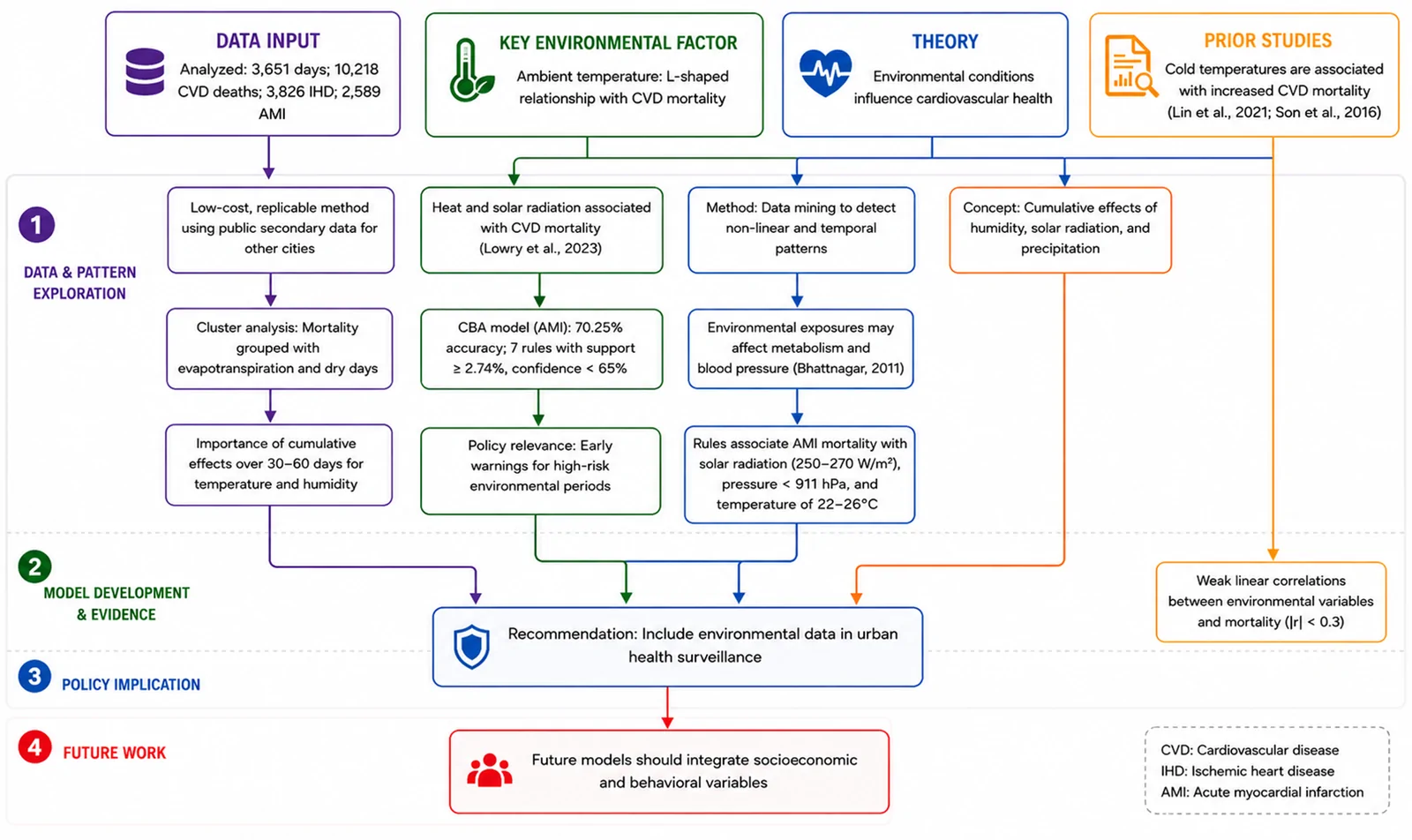
Flowchart with theoretical framework, previous findings, key results, and practical implications of this study.

Among the classification rules, one particularly important condition was the association between low maximum temperatures and increased IHD mortality. Temperatures below 22 °C, observed in the lower tercile, were frequently associated with premature IHD deaths. This is consistent with findings from São Paulo, where higher mortality rates were observed during cold periods [36]. The scientific literature further supports that ambient temperature, circadian rhythm, and seasonal variation influence blood pressure and cardiovascular disease incidence [1], with colder temperatures linked to increased short-term risk of cardiovascular death [5,35,37].

Temporal variables were central to many of the rules identified. In all rules, at least one proposition involved a variable observed over time, highlighting the importance of cumulative environmental exposure. Rather than isolated daily conditions, the classification rules consistently incorporated environmental variables accumulated over periods ranging from 30 to 60 days, suggesting that prolonged exposure better characterized the environmental context associated with premature cardiovascular mortality in this dataset. These findings indicate that cumulative environmental conditions may provide more informative patterns than short-term fluctuations when investigating potential environmental triggers of premature cardiovascular mortality, particularly in the context of climate change [38,39]. Notably, 60-day average humidity was consistently linked to premature IHD deaths, a pattern that may reflect rainfall’s influence, as humidity is highly sensitive to precipitation. Previous studies have also found rainfall to influence cardiovascular mortality [37,40].

Patterns for AMI identified intermediate maximum temperatures (22–26 °C) as one of the environmental conditions present in the classification rules associated with premature deaths. These temperature intervals should not be interpreted as evidence of increased AMI risk within this specific range but rather as conditions identified by the classification model in combination with other environmental variables, such as atmospheric pressure and solar radiation.

An additional limitation of this study is the use of meteorological observations from a single weather station to characterize environmental exposure. Although this approach ensured temporal consistency throughout the study period, it does not capture the spatial heterogeneity of urban microclimates, particularly those associated with urban heat island effects. Consequently, localized differences in thermal exposure across neighborhoods may not be fully represented. Future studies should evaluate the robustness of the identified association rules using multiple meteorological stations or gridded reanalysis datasets, such as ERA5.

Solar radiation and rainfall appeared in rules associated with both the presence and absence of AMI deaths. These variables were identified as components of multivariable environmental patterns rather than as independent predictors of premature AMI mortality. In particular, solar radiation appeared in combination with other environmental variables, such as atmospheric pressure and dew point, indicating that the classification model identified specific combinations of environmental conditions rather than isolated effects of individual variables.

The classifier models developed in this study demonstrate the utility of data mining to investigate the relationship between environmental variables and premature mortality. While broader categories like CVD yielded weaker classification performance, more specific models for IHD and AMI demonstrated better accuracy and interpretability. The AMI model, in particular, generated the largest number of rules, although with moderate confidence values, indicating the complexity of these associations and the need for validation through further studies.

Findings from the IHD model, including the link between cold temperatures and mortality, suggest directions for future research, especially in elderly populations. These insights align with studies demonstrating elevated ischemic heart disease mortality at low temperatures (e.g., 3rd percentile thresholds) and support integrating humidity and rainfall into future CVD-focused research. The observed associations underscore the importance of including vulnerable demographic groups in the design of targeted prevention strategies.

Temporality proved to be a key dimension in this study. Lagged averages allowed for the detection of both immediate and delayed effects of environmental exposures, offering a more comprehensive view of disease dynamics. Future studies should incorporate additional environmental factors such as air pollution, which could further enhance the models’ predictive capacity. Likewise, the inclusion of socioeconomic variables, land use, access to health services, and characteristics of the built environment would offer a more holistic understanding of the environmental determinants of cardiovascular mortality.

An additional limitation of this study is that the classification models were developed without an independent hold-out dataset or cross-validation procedure. Consequently, the reported classification accuracies should be interpreted as exploratory measures of model performance rather than estimates of predictive generalizability. The primary objective of this study was to identify interpretable association patterns and generate hypotheses regarding the relationship between climatic variables and premature cardiovascular mortality. Future studies should externally validate these findings using independent datasets and contemporary validation strategies, such as cross-validation or external testing datasets.

This study demonstrates that the adopted methodology—based on multivariate clustering, classification rule mining, and the use of secondary data—provides a transparent and reproducible framework for exploring associations between environmental conditions and premature cardiovascular mortality in urban areas. Beyond identifying interpretable environmental patterns, the study offers a methodological foundation that may support future investigations incorporating additional environmental, socioeconomic, and health-related variables, as well as external validation using independent datasets.

## Conclusion

This study investigated climate-sensitive temporal patterns associated with premature cardiovascular mortality in a subtropical urban context using an exploratory, data-driven analytical framework. Although linear associations between individual climatic variables and mortality outcomes were weak, the combination of temporal aggregation, clustering, and classification rule mining revealed distinct patterns linked to ischemic heart disease and acute myocardial infarction. These findings highlight the importance of considering cumulative and interacting climatic conditions when examining cardiovascular mortality dynamics.

The results indicate that different cardiovascular outcomes may respond to different climatic profiles. Premature ischemic heart disease mortality was more frequently associated with colder temperatures, elevated long-term humidity, and specific pressure–temperature combinations, whereas premature acute myocardial infarction mortality was linked to intermediate temperature ranges, lower atmospheric pressure, and moderate solar radiation. These heterogeneous patterns reinforce the complexity of climate–health relationships and help explain why traditional linear approaches may fail to capture relevant temporal signals.

Importantly, this study was not designed to estimate causal effects or attributable risks. Instead, it demonstrates the value of an exploratory analytical framework for identifying interpretable associations between climatic conditions and premature cardiovascular mortality and for generating hypotheses to be investigated in future studies. These findings provide a methodological basis for future research incorporating additional environmental, socioeconomic, and spatial variables, as well as external validation using independent datasets.

The methodology applied here is transparent, reproducible, and relies on routinely available mortality and meteorological data, making it suitable for application in other urban settings. This is particularly relevant for low- and middle-income countries, where access to high-resolution exposure data is often limited and climate variability is expected to intensify. Future research integrating additional environmental exposures, socioeconomic characteristics, and spatial information may further enhance the interpretability and applicability of this approach.

## Supporting information

S1 Table Collected (gray row) and Created (non-gray row) Environmental Domain Variables and Mortality for the Research. (DOCX)
