# Supplementary material for: Climate-sensitive patterns of premature cardiovascular mortality in a subtropical city
Source: PLoS One. 2026 Oct 28;21(10):e0357804. doi: 10.1371/journal.pone.0357804 (PMC13716314; doi:10.1371/journal.pone.0357804)
Supplement: Supplementary file 1 [file pone.0357804.s001.docx]

Table S1. Collected (gray row) and Created (non-gray row) Environmental Domain Variables and Mortality for the Research.

| **NATURAL ENVIRONMENT – MODEL INPUT VARIABLE** | | | |
| --- | --- | --- | --- |
| **Variable** | | **Unit** | **Variable name** |
| Sun light | Solar radiation | W/m2 | solar_rad |
|  | Average solar radiation over 2 days | W/m2 | solar_rad_avg_2d |
|  | Average solar radiation over 5 days | W/m2 | solar_rad_avg_5d |
|  | Average solar radiation over 7 days | W/m2 | solar_rad_avg_7d |
|  | Average solar radiation over 30 days | W/m2 | solar_rad_avg_30d |
|  | Average solar radiation over 60 days | W/m2 | solar_rad_avg_60d |
| Temperature | Average temperature | ºC | avg_temp |
|  | Average of Average temperature over 2 days | ºC | avg_temp_2d |
|  | Average of Average temperature over 5 days | ºC | avg_temp_5d |
|  | Average of Average temperature over 7 days | ºC | avg_temp_7d |
|  | Average of Average temperature over 30 days | ºC | avg_temp_30d |
|  | Average of Average temperature over 60 days | ºC | avg_temp_60d |
|  | Minimum temperature | ºC | min_temp |
|  | Average Minimum temperature over 2 days | ºC | min_temp _2d |
|  | Average Minimum temperature over 5 days | ºC | min_temp _5d |
|  | Average Minimum temperature over 7 days | ºC | min_temp _7d |
|  | Average Minimum temperature over 30 days | ºC | min_temp _30d |
|  | Average Minimum temperature over 60 days | ºC | min_temp _60d |
|  | Maximum temperature | ºC | max_temp |
|  | Average Maximum temperature over 2 days | ºC | max_temp_2d |
|  | Average Maximum temperature over 5 days | ºC | max_temp_5d |
|  | Average Maximum temperature over 7 days | ºC | max_temp _7d |
|  | Average Maximum temperature over 30 days | ºC | max_temp _30d |
|  | Average Maximum temperature over 60 days | ºC | max_temp _60d |
|  | Temperature Range | ºC | temp_range |
|  | Average temperature Range over 2 days | ºC | temp_range _2d |
|  | Average temperature Range over 5 days | ºC | temp_range _5d |
|  | Average temperature Range over 7 days | ºC | temp_range _7d |
|  | Average temperature Range over 30 days | ºC | temp_range _30d |
|  | Average temperature Range over 60 days | ºC | temp_range _60d |
| Precipitation | Rain | mm | mm_rain |
|  | Drought | days | drought |
|  | Rainy Days | days | rainy_d |
|  | Rain Accumulated in 2 days | mm | accum_precip_2d |
|  | Rain Accumulated in 5 days | mm | accum_precip_5d |
|  | Rain Accumulated in 7 days | mm | accum_precip_7d |
|  | Rain Accumulated in 30 days | mm | accum_precip_30d |
|  | Rain Accumulated in 60 days | mm | accum_precip_60d |
| Relative humidity | Maximum relative humidity | % | max_humidity |
|  | Average Maximum relative humidity over 2 days | % | max_humidity_2d |
|  | Average Maximum relative humidity over 5 days | % | max_humidity_5d |
|  | Average Maximum relative humidity over 7 days | % | max_humidity_7d |
|  | Average Maximum relative humidity over 30 days | % | max_humidity_30d |
|  | Average Maximum relative humidity over 60 days | % | max_humidity_60d |
|  | Average Relative Humidity | % | avg_humidity |
|  | Average of the daily Average Relative Humidity over 2 days | % | avg_humidity_2d |
|  | Average of the daily Average Relative Humidity over 5 days | % | avg_humidity_5d |
|  | Average of the daily Average Relative Humidity over 7 days | % | avg_humidity_7d |
|  | Average of the daily Average Relative Humidity over 30 days | % | avg_humidity_30d |
|  | Average of the daily Average Relative Humidity over 60 days | % | avg_humidity_60d |
|  | Minimum Relative Humidity | % | min_humidity |
|  | Average Minimum relative humidity over 2 days | % | min_humidity_2d |
|  | Average Minimum relative humidity over 5 days | % | min_humidity_5d |
|  | Average Minimum relative humidity over 7 days | % | min_humidity_7d |
|  | Average Minimum relative humidity over 30 days | % | min_humidity_30d |
|  | Average Minimum relative humidity over 60 days | % | min_humidity_60d |
| Atmospheric humidity | Minimum dew point | ºC | min_dew |
|  | Average Minimum dew point over 2 days | ºC | min_dew_2d |
|  | Average Minimum dew point over 5 days | ºC | min_dew_5d |
|  | Average Minimum dew point over 7 days | ºC | min_dew_7d |
|  | Average Minimum dew point over 30 days | ºC | min_dew_30d |
|  | Average Minimum dew point over 60 days | ºC | min_dew_60d |
|  | Maximum dew point | ºC | max_dew |
|  | Average Maximum dew point over 2 days | ºC | max_dew_2d |
|  | Average Maximum dew point over 5 days | ºC | max_dew_5d |
|  | Average Maximum dew point over 7 days | ºC | max_dew_7d |
|  | Average Maximum dew point over 30 days | ºC | max_dew_30d |
|  | Average Maximum dew point over 60 days | ºC | max_dew_60d |
| Climate Humidity | Potential Evapotranspiration | mm | pot_evapotrans |
|  | Average Potential Evapotranspiration over 2 days | mm | pot_evapotrans_2d |
|  | Average Potential Evapotranspiration over 5 days | mm | pot_evapotrans_5d |
|  | Average Potential Evapotranspiration over 7 days | mm | pot_evapotrans_7d |
|  | Average Potential Evapotranspiration over 30 days | mm | pot_evapotrans_30d |
|  | Average Potential Evapotranspiration over 60 days | mm | pot_evapotrans_60d |
| Atmospheric pressure | Average atmospheric pressure | hPa | avg_pressure |
|  | Average of Average atmospheric pressure over 2 days | hPa | avg_pressure_2d |
|  | Average of Average atmospheric pressure over 5 days | hPa | avg_pressure_5d |
|  | Average of Average atmospheric pressure over 7 days | hPa | avg_pressure_7d |
|  | Average of Average atmospheric pressure over 30 days | hPa | avg_pressure_30d |
|  | Average of Average atmospheric pressure over 60 days | hPa | avg_pressure_60d |
|  | Minimum atmospheric pressure | hPa | min_pressure |
|  | Average Minimum atmospheric pressure over 2 days | hPa | min_pressure_2d |
|  | Average Minimum atmospheric pressure over 5 days | hPa | min_pressure_5d |
|  | Average Minimum atmospheric pressure over 7 days | hPa | min_pressure_7d |
|  | Average Minimum atmospheric pressure over 30 days | hPa | min_pressure_30d |
|  | Average Minimum atmospheric pressure over 60 days | hPa | min_pressure_60d |
|  | Maximum atmospheric pressure | hPa | max_pressure |
|  | Average Maximum atmospheric pressure over 2 days | hPa | max_pressure_2d |
|  | Average Maximum atmospheric pressure over 5 days | hPa | max_pressure_5d |
|  | Average Maximum atmospheric pressure over 7 days | hPa | max_pressure_7d |
|  | Average Maximum atmospheric pressure over 30 days | hPa | max_pressure_30d |
|  | Average Maximum atmospheric pressure over 60 days | hPa | max_pressure_60d |
| Wind | Average speed | km/h | avg_wind |
|  | Average of Average speed over 2 days | km/h | avg_wind_2d |
|  | Average of Average speed over 5 days | km/h | avg_wind_5d |
|  | Average of Average speed over 7 days | km/h | avg_wind_7d |
|  | Average of Average speed over 30 days | km/h | avg_wind_30d |
|  | Average of Average speed over 60 days | km/h | avg_wind_60d |
|  | Maximum Gust Speed | km/h | max_wind_gust |
|  | Average Maximum Gust Speed over 2 days | km/h | max_wind_gust_2d |
|  | Average Maximum Gust Speed over 5 days | km/h | max_wind_gust_5d |
|  | Average Maximum Gust Speed over 7 days | km/h | max_wind_gust_7d |
|  | Average Maximum Gust Speed over 30 days | km/h | max_wind_gust_30d |
|  | Average Maximum Gust Speed over 60 days | km/h | max_wind_gust_60d |
|  | Direction | Categorical variable | direction |
| Seasonality | Seasons | Categorical variable | seasons |
|  | Month | Categorical variable | month |
|  | Whether weekend or weekday | Categorical variable | weekend |
| **MORTALITY – MODEL OUTPUT VARIABLE \| MULTIVARIATE ANALYSIS** | | | |
| **CVD** | | **Unit** | **Variable name** |
| Mortality | Number of deaths per day | number | count_cases_CVD |
| **IHD** |  |  |  |
| Mortality | Number of deaths per day | number | count_cases_IHD |
| **AMI** |  |  |  |
| Mortality | Number of deaths per day | number | count_cases_AMI |
| **MORTALITY – MODEL OUTPUT VARIABLE \| CBA** | | | |
| **CVD** |  | **Unit** | **Variable name** |
| Mortality | No deaths; 1-4 deaths; more than 4 deaths | category | count_cases_CVD |
| **IHD** |  |  |  |
| Mortality | Whether or not there were deaths on the day | category | count_cases_IHD |
| **AMI** |  |  |  |
| Mortality | Whether or not there were deaths on the day | category | count_cases_AMI |
