# Supplementary material for: Climate-sensitive patterns of premature cardiovascular mortality in a subtropical city
Source: PLoS One. 2026 Oct 28;21(10):e0357804. doi: 10.1371/journal.pone.0357804 (PMC13716314; doi:10.1371/journal.pone.0357804)
Supplement: Supplementary file 2 [file pone.0357804.s003.docx]

# Response to Reviewers

**Manuscript Title:** Climate-sensitive patterns of premature cardiovascular mortality in a subtropical city
**Journal:** Plos One
**Manuscript ID:** PONE-D-26-00863

Dear Editor and Reviewers,

We sincerely thank the Editor and the Reviewers for the careful evaluation of our manuscript and for the valuable comments and suggestions. We have carefully addressed every point raised. All changes made in the revised manuscript are described below, and the corresponding modifications have been highlighted in the revised version.

## Reviewer 1

| **Reviewer** | **Comment** | **Response** | **Changes in manuscript** |
| --- | --- | --- | --- |
| Reviewer 1 | Innovation in Methodology: The great success of the study lies in replacing traditional linear statistics with Data Mining (Artificial Intelligence). Since climate and human health are related in a complex and non-linear way, the use of algorithms such as K-means and CBA proved to be the right choice for finding patterns that would be missed in common analyses. | We sincerely thank the reviewer for the positive assessment of the methodological approach. We strengthened the Introduction and Discussion to better explain why clustering and association rule mining were selected as complementary exploratory techniques for identifying nonlinear climate–health relationships. We also clarified that the proposed framework is intended for hypothesis generation rather than causal inference. | Introduction; Discussion (methodological rationale strengthened). |
| Reviewer 1 | Importance of Lags (Time Delays): The creation of 90 lag variables (cumulative effects of 2 to 60 days) was a crucial methodological success. The impact of climate on the human body is rarely immediate; the study proves that cardiovascular stress is a reflection of meteorological conditions that linger or vary over days and weeks. | We appreciate this observation. We expanded the Methods section to explain the rationale for creating lagged variables (2, 5, 7, 30 and 60 days) and discussed how cumulative environmental exposure may better represent delayed physiological responses than same-day measurements alone. | Methods – Data Preparation; Discussion (lag interpretation). |
| Reviewer 1 | Dynamics of Results (Climate and the Heart):The study managed to separate the "atmospheric behavior" of two serious conditions:  Ischemic Heart Disease (IHD): Showed to be linked to chronic stress from prolonged cold and high humidity.  Acute Myocardial Infarction (AMI): Showed to be more acute, associated with lower atmospheric pressure (common in abrupt weather changes and cold fronts), which can destabilize the circulatory system. | Thank you for this insightful observation. We agree that the classification models identified distinct environmental patterns associated with IHD and AMI. To better highlight this finding, we revised the Discussion to more clearly distinguish the climatic profiles identified for each outcome, emphasizing that the models revealed different combinations of environmental conditions associated with IHD and AMI while interpreting these patterns as exploratory associations rather than causal relationships. | Discussion (comparison between IHD and AMI expanded). |
| Reviewer 1 | Incomplete data (AMI section): The final section on AMI (which had the best accuracy, 70.25%) was cut from the presented text and needs to be detailed. | We agree that the AMI results required further explanation. The Results section describing the AMI classifier was expanded to provide a more detailed presentation of the identified classification rules, their confidence values, and the environmental conditions associated with each rule. The Discussion was also revised to better contextualize these findings while interpreting them as exploratory multivariable patterns rather than evidence of independent climatic effects. | Results (AMI subsection expanded); Discussion revised. |
| Reviewer 1 | Missing variables (air pollution): The model would greatly benefit from including air pollution, which tends to increase in winter along with heart disease. | We agree that air pollution is an important determinant of cardiovascular health. Unfortunately, complete daily air-quality data covering the entire study period were not available. We now explicitly acknowledge this limitation and identify the inclusion of air pollution as a priority for future studies. | Discussion (Limitations); Conclusions (future research). |
| Reviewer 1 | Practical conclusion / Early warning systems: The article is excellent for generating hypotheses and has immediate real-world application. These weather patterns can be used by governments to create Early Warning Systems, allowing hospitals and vulnerable groups to prepare days before a critical weather event. | We appreciate this thoughtful suggestion. We revised the Discussion and Conclusions to emphasize the methodological contribution of the proposed framework as an exploratory approach for identifying climate-sensitive associations and generating hypotheses. To avoid overstating the current findings, we no longer suggest immediate application to operational early warning systems and instead highlight the need for additional variables and external validation before practical implementation. | Discussion final paragraphs; Conclusion revised. |

## Reviewer 2

| **Reviewer** | **Comment** | **Response** | **Changes in manuscript** |
| --- | --- | --- | --- |
| Reviewer 2 | The manuscript titled “Climate-sensitive patterns of premature cardiovascular mortality in a subtropical city” investigates associations between daily climatic conditions and premature mortality from CVD, IHD and AMI in Curitiba, Brazil, over the period spanning 2010-2019. The authors present an exploratory, data-driven framework which combines multivariate analysis, k-means and hierarchical clustering, and classification-based association rule mining. Even though the research is interesting and is based on a substantial dataset (spans 10 years), the manuscript has several significant methodological weaknesses that must be addressed. | We sincerely thank the reviewer for the thorough and constructive evaluation of our manuscript. We appreciate the recognition of the relevance of the research question and the value of the 10-year dataset. We carefully considered all methodological concerns and substantially revised the manuscript accordingly. The major revisions include: (i) strengthening the methodological rationale for the exploratory analytical framework; (ii) expanding the description and justification of the lagged variables and clustering procedures; (iii) clarifying the interpretation of the classification models as exploratory rather than predictive or causal; (iv) revising the Discussion and Conclusion to avoid unsupported interpretations and overstatement of the findings; and (v) explicitly acknowledging the study limitations, including the lack of external validation and additional environmental variables. We believe these revisions have substantially improved the clarity, rigor, and scientific interpretation of the study, and we sincerely appreciate the reviewer's valuable comments. | The manuscript was comprehensively revised in response to the reviewer's comments. Revisions were made throughout the Introduction, Methods, Results, Discussion, and Conclusion to strengthen the methodological rationale, clarify the interpretation of the analytical framework, improve the presentation of the results, avoid unsupported interpretations, and explicitly acknowledge the study limitations. Specific changes are detailed in the responses to each comment below. |
| Reviewer 2 | 1. The first four paragraphs of the introduction are short, repetitive and light on references (only 1 is used). I suggest to combine them in a single concise paragraph with more substantial information. | Thank you for this valuable suggestion. We substantially revised the opening section of the Introduction by merging the first four short paragraphs into a single, more concise paragraph. We removed repetitive statements, strengthened the scientific rationale, and incorporated additional references to provide broader support for the relationship between environmental exposures and cardiovascular disease before introducing the specific evidence on temperature and climate. | The first four paragraphs of the Introduction were consolidated into a single paragraph, redundant descriptions of the natural environment and geosphere were removed, and additional references on environmental determinants of cardiovascular disease and climate-health interactions were incorporated. This revision improves readability and provides a stronger background for the study. |
| Reviewer 2 | 2. Lines 88-89: How has climate change affected urban temperatures specifically? | Thank you for this suggestion. We agree that the original statement was too general. We revised the text to explain the mechanisms through which climate change and urbanization affect urban temperatures, including the increased frequency and intensity of heat events and the amplification of warming through the urban heat island effect, vegetation loss, and heat storage in impervious surfaces. | The description of climate change impacts on urban temperatures was expanded to clarify the mechanisms linking climate change, urbanization, and increases in average and minimum temperatures before discussing their association with cardiovascular mortality. |
| Reviewer 2 | 3. The paragraph that spans lines 94-103 makes significant statements but only refers to the same paper again (ref. 1). | Thank you for this observation. We agree that the paragraph relied excessively on a single reference. We revised the text by incorporating additional references supporting the specific statements regarding seasonal variation, blood pressure, cardiovascular mortality, temperature-related cardiovascular events, and the expected impacts of climate change on cardiovascular health. | Additional references were incorporated throughout the paragraph to support the different mechanisms and epidemiological evidence discussed, reducing reliance on a single review article and providing a more balanced overview of the current literature. |
| Reviewer 2 | 4. Lines 106-108: Be more specific about past research. What does the literature state about this association? which previous studies, what were the outcomes of these studies? | Thank you for this suggestion. We expanded this section by providing specific examples from previous studies. The revised text now summarizes how earlier research has applied data mining and machine learning to cardiovascular disease prediction and environmental epidemiology, highlighting their main findings and clarifying how our study differs by focusing on climate-sensitive temporal patterns using multivariate clustering and associative classification rule mining. | The Introduction was revised to include specific examples of previous studies, their analytical approaches, and their main findings, providing a clearer rationale for the proposed methodology and its contribution relative to the existing literature. |
| Reviewer 2 | 5. Line 110, Recent studies reinforce... Which recent studies? The same reference is used quite a lot in the introduction ([1]). | Thank you for pointing this out. We agree that the previous wording relied excessively on Reference 1 and incorrectly referred to "recent studies." The paragraph was revised to incorporate additional and more recent references specifically addressing the relationship between sunlight exposure, environmental conditions, and cardiovascular health. We also replaced the general statement with a more evidence-based description of the reported associations. | The paragraph discussing sunlight exposure and cardiovascular health was revised to include additional recent references and to describe the specific evidence supporting the association between environmental exposures and cardiovascular outcomes, thereby reducing reliance on Reference 1. |
| Reviewer 2 | 6. Sentence in line 113: Need to expand this. Which previous studies, which data mining techniques etc..? | Thank you for this suggestion. We expanded this section by describing the previous studies that served as the methodological basis for our work. The revised text now specifies the environmental and urban applications in which data mining techniques were successfully applied, including landslide prediction, wildfire occurrence, urban planning, respiratory morbidity, and water quality monitoring. We also clarified the analytical techniques employed and how these studies motivated the methodology adopted in the present research. | The Introduction was revised to summarize the objectives and main outcomes of the previous studies cited in References 15–20, clarifying the data mining techniques employed and strengthening the rationale for applying a similar analytical framework to climate-sensitive cardiovascular mortality. |
| Reviewer 2 | 7. Line 126, “Current findings indicate...”, need to summarise current findings with appropriate citations. | Thank you for this comment. We agree that the original sentence was too generic. During the revision of the Introduction, the original statement was replaced by a more specific summary of previous studies. The revised text now identifies the main data mining techniques used for cardiovascular disease prediction and summarizes their reported contributions, supported by appropriate citations. | The original sentence was replaced by a more detailed description of previous studies on cardiovascular disease prediction, specifying the analytical techniques employed (e.g., decision trees, support vector machines, artificial neural networks, and ensemble methods) and their reported predictive performance, with supporting references. |
| Reviewer 2 | 8. Line 127, “New approaches...” which new approaches? what were the findings? | Thank you for this observation. We clarified what we meant by "new approaches" by explicitly identifying the current limitation of existing prediction models. The revised text now explains that most machine learning studies rely predominantly on clinical and demographic variables, with limited incorporation of environmental exposures, and highlights the need for approaches that integrate environmental and temporal information into cardiovascular disease prediction. | The sentence was revised to specify the limitations of existing machine learning approaches and to clarify that the proposed methodological advancement consists of integrating environmental and temporal variables with conventional cardiovascular risk factors, thereby providing a clearer rationale for the present study. |
| Reviewer 2 | 9. Line 135, “..allows for an understanding..”, remove “an” | Thank you for identifying this grammatical issue. The sentence was revised to improve clarity and grammatical correctness. | The expression "allows for an understanding" was replaced with "enables a better understanding of" to improve readability and conform to standard scientific English. |
| Reviewer 2 | 1. Paragraphs spanning lines 139-153 belong in the introduction. | Thank you for this suggestion. We agree that these paragraphs provide contextual information about the study area rather than methodological procedures. Therefore, they were relocated from the beginning of the Methods section to the end of the Introduction to improve the organization and flow of the manuscript. | The paragraphs describing the study area, the rationale for selecting Curitiba, and its relevance to the research were moved from the beginning of the Methods section to the end of the Introduction. The Methods section now begins with a brief overview of the methodological approach, followed by the *Data Acquisition* subsection. |
| Reviewer 2 | 2. Line 167, SMS was not defined before | Thank you for pointing this out. We have defined the acronym "SMS" at its first occurrence in the manuscript. | The first mention of SMS was revised to "The Municipal Health Secretariat of Curitiba (Secretaria Municipal da Saúde – SMS)" to define the acronym before its subsequent use. |
| Reviewer 2 | 3. Line 180. Only a single station is used. While this is not uncommon in similar studies, the authors make no effort to describe the relevancy of this station to the rest of the city. Where is it located with respect with the city etc. The authors themselves cite evidence that heat islands were documented in Curitiba in 2019, with temperatures in the urban core exceeding 29°C and temperatures in vegetated areas up to 10°C lower. This documented within-city thermal heterogeneity means that the single-station exposure values may systematically misclassify the actual temperature experienced by residents in different neighbourhoods. This is particularly consequential for AMI, where the identified temperature range of 22–26°C spans only four degrees, a range fully encompassed by the within-city variability the authors acknowledge. The authors should also consider whether multi-station data (additional SIMEPAR stations in Curitiba's metropolitan area) or gridded reanalysis products (e.g., ERA5) could be used to test the sensitivity of the classification rules to the choice of exposure data. | Thank you for this thoughtful and constructive comment. We agree that the spatial representativeness of meteorological exposure deserves further clarification. We have revised the Methods section to provide additional information on the meteorological station used and to explain the rationale for its selection. The station was chosen because it provides continuous, quality-controlled observations covering the entire study period (2010–2019), ensuring temporal consistency across all meteorological variables analyzed. We also acknowledge that the use of a single weather station does not capture the spatial heterogeneity associated with urban heat island effects and may not fully represent localized microclimatic conditions across Curitiba. Accordingly, we have expanded the Discussion to explicitly recognize this limitation and to recommend that future studies evaluate the robustness of the identified association rules using multiple meteorological stations or gridded reanalysis datasets, such as ERA5. | The Methods section was revised to justify the selection of the meteorological station and clarify its role in characterizing city-scale temporal variability. In addition, the Discussion was expanded to acknowledge the limitations associated with using a single weather station and to recommend the use of multiple meteorological stations or gridded reanalysis products (e.g., ERA5) in future studies. |
| Reviewer 2 | 4. What is the justification for the use of these variables (Table 1). | Thank you for this important comment. We agree that the rationale for selecting the environmental variables should be made explicit. We revised the introductory section of the Methods to clarify that the selected variables were based on previous evidence demonstrating the influence of multiple meteorological factors on cardiovascular outcomes. Appropriate references supporting the inclusion of these variables were also added. | The introductory paragraph of the Methods was revised to explicitly justify the selection of the environmental variables included in Table 1 and to cite the literature supporting their relevance to cardiovascular mortality. |
| Reviewer 2 | 5. The paragraph spanning lines 194-197 is redundant, remove it. | Thank you for this suggestion. We agree that this paragraph was redundant and have removed it to improve the conciseness of the Methods section. | The redundant paragraph describing the general rationale for generating additional variables during data preparation was removed. The section now proceeds directly to the description of the variables created and the preprocessing procedures. |
| Reviewer 2 | 6. What is the justification for using lag times up to 60 days? | Thank you for this important comment. We agree that the rationale for selecting the lag periods should be clarified. The lag periods (2, 5, 7, 30, and 60 days) were established as methodological choices within the exploratory data mining framework to evaluate different temporal aggregation windows, rather than to represent predefined biological exposure periods. This strategy was adopted to allow the algorithm to identify the temporal scales most strongly associated with cardiovascular mortality while capturing both short-term and cumulative environmental exposures. We have revised the Methods section accordingly and added references supporting the relevance of meteorological conditions to cardiovascular outcomes. | The Methods section was revised to explain the rationale for selecting the lag periods (2, 5, 7, 30, and 60 days). The revised text clarifies that these intervals were adopted as methodological choices within the exploratory data mining approach to evaluate multiple temporal aggregation windows and includes references supporting the relationship between meteorological conditions and cardiovascular outcomes. |
| Reviewer 2 | 7. Lines 206-208, how were these calculated? | Thank you for this comment. We agree that the discretization procedure required further clarification. We have revised the Methods section to explain how the tercile cut-off values were calculated. Specifically, the thresholds were determined independently for each continuous variable using its empirical distribution, with the 33.3rd and 66.7th percentiles defining the boundaries between the low, intermediate, and high categories. | The Methods section was revised to describe the calculation of the tercile cut-off values used during the discretization stage, thereby improving the reproducibility of the analytical procedure. |
| Reviewer 2 | 8. Discretization is an important and common preprocessing step, but it is not universally necessary for all data mining tasks. Many algorithms (e.g., linear regression, support vector machines, neural networks) work directly with continuous variables without requiring discretization. | Thank you for this important clarification. We agree that discretization is not universally required for all data mining techniques. In our study, however, discretization was specifically required because the Classification Based on Associations (CBA) algorithm is based on association rule mining and operates on categorical attributes. We have revised the Methods section to clarify that discretization was performed to meet the requirements of the CBA algorithm rather than as a general requirement for data mining. | The Methods section was revised to clarify that discretization was specifically performed to enable the application of the Classification Based on Associations (CBA) algorithm, which requires categorical variables for association rule generation and classification. |
| Reviewer 2 | 9. In general this section contains repetitive statements and not nearly enough details to reproduce the experiment. (Data Preparation section). CBA is quite dated. why use this methods and not something more contemporary? | Thank you for this valuable comment. We agree that the original Data Preparation section contained unnecessary repetition and required additional methodological details to improve reproducibility. Accordingly, we revised this section by removing redundant statements, reorganizing the preprocessing workflow, and providing a more detailed description of dataset integration, temporal lag generation, derived variables, and the discretization procedure.  Regarding the use of the Classification Based on Associations (CBA) algorithm, we acknowledge that more recent machine learning methods are available. However, the primary objective of this study was not to maximize predictive performance, but to identify transparent and interpretable relationships between environmental variables and premature cardiovascular mortality. For this reason, CBA was selected because it generates explicit classification rules that facilitate knowledge discovery and support the interpretation of environmental patterns in a public health context. This rationale has now been explicitly incorporated into the manuscript. | The Data Preparation section was revised to remove repetitive statements and include additional methodological details regarding dataset integration, lag generation, derived variables, and the discretization procedure, thereby improving the reproducibility of the study. In addition, the Data Modeling section was expanded to clarify the rationale for selecting the CBA algorithm, emphasizing that its choice was based on the study's objective of generating transparent and interpretable classification rules rather than maximizing predictive performance. |
| Reviewer 2 | 10. e.g. random forest, xgboost. These could be wrapped in a SHAP-model for further explainability etc.. | Thank you for this valuable suggestion. We agree that more recent machine learning algorithms, such as Random Forest and XGBoost, combined with explainability frameworks such as SHAP, are powerful approaches for predictive modeling and model interpretation. However, the primary objective of this study was not to maximize predictive performance, but to identify transparent and directly interpretable associations between environmental variables and premature cardiovascular mortality. For this reason, we selected the Classification Based on Associations (CBA) algorithm, which directly generates human-readable classification rules without requiring post hoc interpretation techniques. To clarify this rationale, we expanded the Data Modeling section of the manuscript. | The Data Modeling section was revised to clarify the rationale for selecting the CBA algorithm, emphasizing that its selection was based on the study's objective of generating explicit and interpretable classification rules rather than maximizing predictive performance. |
| Reviewer 2 | 11. Line 254, why different thresholds for CVD deaths? The authors do not discuss this. | Thank you for this important comment. We agree that the rationale for the different outcome thresholds was not sufficiently explicit. We have revised the manuscript to clarify that the CVD categories were defined according to the tercile distribution of the daily cardiovascular mortality counts. Because the daily frequencies of IHD and AMI deaths were considerably lower, these outcomes were modeled as binary variables (presence/absence of deaths). | The "Classifier Targets" subsection was revised to clarify that the thresholds adopted for CVD deaths were based on the tercile distribution of the observed daily counts, whereas IHD and AMI were analyzed as binary outcomes due to their lower daily frequencies. |
| Reviewer 2 | 12. Line 257, repetitive statement, remove. | Thank you for this observation. We agree that this statement was redundant because the study population and study period had already been described in the Data Acquisition section. The sentence has been removed to improve the conciseness of the manuscript. | The redundant sentence following the definition of the classifier targets was removed. |
| Reviewer 2 | 13. Lines 274-275: why different pruning control parameters? | Thank you for this important comment. We agree that the rationale for adopting different pruning control parameters was not sufficiently explained. We have revised the manuscript to clarify that pruning settings were empirically selected after preliminary testing to maximize classification performance for each outcome. The IHD classifier achieved better performance with pruning enabled, whereas the CVD and AMI classifiers performed better without pruning. | The Data Modeling section was revised to explain that the pruning control parameters were empirically selected during preliminary model development according to the classification performance achieved for each outcome. |
| Reviewer 2 | 14. For the model evaluation, no held-out portion, no cross-validation? How can these be trusted that it's not overfitting? Results | Thank you for this important methodological comment. We agree that the absence of an independent hold-out dataset or cross-validation procedure limits the assessment of the models' predictive generalizability. The primary objective of this study, however, was not to develop predictive models but to identify interpretable association patterns and generate hypotheses regarding the relationship between climatic variables and premature cardiovascular mortality. To clarify this, we revised the Data Modeling section to explicitly state the exploratory purpose of the classification models and the rationale for selecting the CBA algorithm. In addition, we expanded the Discussion to acknowledge the absence of external validation as a limitation and to emphasize that future studies should validate the proposed approach using independent datasets and contemporary validation strategies, such as cross-validation or external testing datasets. | The Data Modeling section was revised to clarify that the classification models were designed for exploratory pattern discovery and hypothesis generation rather than predictive model development, justifying the use of the CBA algorithm. The Discussion was also expanded to acknowledge the lack of an independent validation procedure as a study limitation and to recommend external validation in future research. |
| Reviewer 2 | 1. The authors should explicitly state: - Why the 90 derived/lagged variables were excluded from the correlation matrix - Whether this selective inclusion could have biased the variable relationships presented | Thank you for this important observation. We have revised the manuscript to clarify that the correlation matrix was intentionally constructed using only the original meteorological variables. The derived (lagged and accumulated) variables were generated subsequently during the feature engineering stage specifically for the classification models and were therefore not included in the descriptive correlation analysis. Including these variables would have introduced multiple highly correlated representations of the same environmental measurements, making the correlation matrix more difficult to interpret. Consequently, their exclusion did not bias the relationships presented but rather preserved the descriptive purpose of the analysis. | The Data Preparation section was revised to explicitly state that the correlation matrix included only the original meteorological variables because the lagged and accumulated variables were generated later for feature engineering and classification modeling. The rationale for excluding these derived variables from the correlation analysis was also added. |
| Reviewer 2 | 2. Fig 1: The authors acknowledge the high inter-variable correlations (r > 0.7) in a single sentence, dismissing them as physically expected, but they provide no methodological response to this finding. They do not apply dimensionality reduction, variable selection, or any correction before feeding all variables into the clustering and classification models. Also for the modelling part K-means uses Euclidean distances, which are distorted by redundant variables. Highly correlated variables effectively get double- or triple-weighted in the distance calculation. For instance, including avg_pressure, min_pressure, and max_pressure (all r > 0.97 ) means atmospheric pressure dominates cluster formation disproportionately. For the regression-based modelling part Multicollinearity inflates standard errors, destabilizes coefficient estimates, and reduces statistical power, making it difficult to isolate the independent contribution of each variable. For example, if avg_temp, min_temp, and max_temp (all r > 0.89 with each other ) are all included in a regression, their individual coefficients become unreliable. For the CBA classification model too, feeding highly correlated discretised variables (e.g., avg_pressure, min_pressure, max_pressure all in terciles) means the algorithm is effectively sampling the same underlying signal three times when generating rules, inflating apparent rule support. | Thank you for this thoughtful methodological comment. We agree that several meteorological variables exhibit high correlations because they represent different summaries (minimum, average, and maximum values) of the same environmental phenomenon. We would first like to clarify that this study did not employ regression-based models; therefore, concerns regarding coefficient instability and inflated standard errors are not directly applicable. Regarding the clustering analysis, we acknowledge that correlated variables may influence Euclidean distance calculations. However, clustering was used exclusively as an exploratory technique to identify overall similarity patterns and was not employed for variable selection or statistical inference. For the associative classification analysis, although correlated variables may generate overlapping candidate rules, the CBA algorithm applies rule selection mechanisms based on minimum support, confidence thresholds, rule ranking, and pruning, which reduce redundancy in the final classifier. To clarify these methodological aspects, we have expanded the Discussion accordingly. | The Discussion was revised to clarify the exploratory purpose of the clustering analyses, explain why correlated meteorological variables were retained, distinguish the adopted methodology from regression-based approaches, and discuss how the CBA rule selection process mitigates the influence of redundant candidate rules. |
| Reviewer 2 | 3. why all 104 variables are used here and not in the previous experiment? (line 305) | Thank you for this important observation. We agree that the distinction between the variables used in the correlation analysis and those used in the clustering analysis was not sufficiently clear. The correlation matrix was intentionally restricted to the original meteorological variables because its objective was to describe the linear relationships among the measured environmental variables. In contrast, the clustering analysis was performed using the complete set of 104 engineered variables, including lagged and accumulated measures generated during the feature engineering stage. These derived variables were specifically created to capture delayed and cumulative environmental effects that could not be represented by the original measurements alone. We have revised the manuscript to explicitly clarify this methodological distinction. | The Results section was revised to clarify that the correlation analysis was restricted to the original meteorological variables for descriptive purposes, whereas the clustering analysis included the complete set of engineered variables (104 variables), including lagged and accumulated measures, to explore similarity patterns across the full feature space generated during data preparation. |
| Reviewer 2 | 4. The sentences in lines 320-321 is repetitive and redundant, remove. | Thank you for this observation. We agree that the identified sentence was repetitive and did not add new information to the interpretation of the results. Therefore, it has been removed to improve the clarity and conciseness of the manuscript. | The redundant sentence at the end of the dendrogram description was removed from the Results section to improve readability and avoid repetition. |
| Reviewer 2 | 5. Fig 2: Caption states 104 whereas diagram title 107. which one is correct? | Thank you for identifying this inconsistency. The dendrogram includes a total of 107 variables, comprising 104 numerical environmental variables and three dependent variables (count_cases_CVD, count_cases_IHD, and count_cases_AMI). The figure title was correct, whereas the caption mistakenly referred only to the environmental variables. We have corrected the figure caption accordingly. | The caption of Figure 2 was revised to correctly indicate that the dendrogram includes 107 variables, consisting of 104 numerical environmental variables and the three dependent mortality variables. |
| Reviewer 2 | 6. Fig 2: the x-axis is not readable | Thank you for this observation. We agree that the x-axis labels in Figure 2 were difficult to read at the published scale. To improve readability, the figure has been regenerated with higher resolution and enlarged x-axis labels, ensuring that the variable names are legible in the final version of the manuscript. | Figure 2 was regenerated with improved resolution and enlarged x-axis labels to enhance readability while preserving the original clustering results. |
| Reviewer 2 | 7. Line 328, “It was identified..” how was this identified? Why not use standard methods such as the elbow method or the silhouette scores method? | Thank you for this valuable comment. We agree that the original wording did not clearly explain how the cluster patterns were identified. The hierarchical clustering results were interpreted through visual inspection of the dendrogram, as the objective of this analysis was exploratory and aimed to identify similarity patterns among variables rather than determine an optimal cluster partition. Consequently, formal cluster validation methods, such as the elbow method or silhouette analysis, were not applied. We have revised the Methods section to explicitly describe this procedure and expanded the Discussion to acknowledge this methodological choice and its implications. | The Methods section was revised to clarify that the hierarchical clustering results were interpreted through visual inspection of the dendrogram for exploratory purposes. The manuscript now explicitly states that formal cluster validation methods, including the elbow method and silhouette analysis, were not applied because the clustering analysis was intended to explore similarity patterns rather than optimize the number of clusters. The Discussion was also updated to acknowledge this methodological choice as a limitation and to suggest the application of formal validation metrics in future studies. |
| Reviewer 2 | 8. Table 2: Cluster 2 is oddly heterogeneous, discuss. is the accumulated precipitation over the past 60 days a really relevant feature? | Thank you for this insightful comment. We agree that Cluster 2 appears more heterogeneous than the remaining clusters, as it groups accumulated precipitation over 60 days with solar radiation variables despite representing different meteorological processes. We have expanded the Discussion to clarify that this reflects the unsupervised nature of k-means clustering, which groups variables according to statistical similarity rather than their physical interpretation. We also clarify that this result should be interpreted as a statistical association rather than evidence of a direct environmental relationship. Furthermore, we emphasize that accumulated precipitation over 60 days did not emerge as one of the most influential variables in the final classification models, suggesting a limited contribution to the prediction of premature cardiovascular mortality. | A new paragraph was added to the Discussion acknowledging the heterogeneous composition of Cluster 2. The revised text explains that heterogeneous groupings may occur because k-means clustering is based on statistical similarity rather than physical interpretation and clarifies that accumulated precipitation over 60 days did not emerge as one of the most influential variables in the final classification models. |
| Reviewer 2 | 9. Lines 348-349, “The model generated...” then why were these included? Have you tried running the model with a simpler rule (like the IHD and AMI cases)? | Thank you for this important observation. We agree that the original text did not sufficiently justify the inclusion of the CVD rules that did not meet the predefined minimum support and confidence thresholds. These rules were included solely for completeness and to facilitate comparison with the IHD and AMI classification models, rather than being interpreted as robust classification patterns. We have revised the manuscript to clarify this rationale. We did not perform an additional analysis using a simplified rule set because the objective of this study was to evaluate the classifier under the same predefined criteria for all three outcomes (CVD, IHD, and AMI), allowing direct methodological comparison across the models. | The Results section was revised to clarify that the three CVD rules presented in Table 3 were included only because they were the closest to meeting the predefined support and confidence thresholds. The revised text explicitly states that these rules are shown for completeness and comparison with the IHD and AMI models and should not be interpreted as robust classification rules. |
| Reviewer 2 | 10. In table 3 the CVD rules are all below the thresholds set by the authors. Why include them? | Thank you for this important comment. We agree that presenting CVD rules below the predefined support and confidence thresholds could be misleading. These rules were included solely for completeness and to facilitate comparison with the IHD and AMI classification results. They were not considered valid classification rules nor interpreted as robust predictive patterns. To avoid any ambiguity, we have revised the Results section to explicitly clarify this rationale and updated the caption of Table 3 to state that the CVD rules did not meet the predefined support and confidence thresholds and are presented exclusively for descriptive and comparative purposes. | The Results section was revised to clarify that the CVD rules presented in Table 3 did not meet the predefined support and confidence thresholds and are included only for descriptive completeness and comparison with the IHD and AMI models. In addition, the caption of Table 3 was updated to explicitly indicate that these rules are shown for descriptive and comparative purposes only and should not be interpreted as robust classification rules. |
| Reviewer 2 | 11. Line 363, “demonstrating its robustness..” How is this demonstrating its robustness? | Thank you for this important comment. We agree that the original wording overstated the interpretation of the results by referring to the model's "robustness," which was not directly demonstrated by the analyses performed. Our intention was to emphasize the model's ability to identify interpretable patterns associated with IHD mortality rather than to claim predictive robustness or generalizability. We have therefore revised the text to more accurately reflect the scope of the findings, replacing the unsupported statement with language describing the model's ability to identify interpretable patterns and its potential to provide new insights into the relationship between environmental conditions and IHD mortality. | The Discussion section was revised by replacing the statement that the model demonstrated "robustness" with wording indicating that the model identified interpretable patterns associated with IHD mortality. The revised text also emphasizes the model's potential to provide new insights into the relationship between environmental conditions and IHD mortality, avoiding unsupported claims regarding its robustness or generalizability. |
| Reviewer 2 | 12. Line 375, show should be shows | Thank you for pointing out this grammatical issue. During the revision process, this sentence was rewritten in response to other reviewer comments. The revised wording resolves the grammatical error while also improving the clarity and accuracy of the statement. | The sentence was revised as part of the manuscript revision, correcting the grammatical issue and improving its clarity in accordance with the other changes made to the Discussion section. |
| Reviewer 2 | 13. Line 386 “Nonetheless, recognizing this limitation...” I disagree with this statement. The results presented for the classification model do not seem to be robust, so how can they be used in comparisons in future studies? Discussion | Thank you for this valuable comment. We agree that the original wording overstated the implications of the classification results. Our intention was not to suggest that the results themselves are sufficiently robust to support direct comparisons with future studies, but rather that the proposed methodological approach may be useful for future investigations. We have therefore revised the text to clarify this point and to emphasize that further studies incorporating additional datasets and external validation are needed before broader application of the approach. | The sentence stating that the results could be used for comparisons with future studies was revised. The updated text now clarifies that the methodological approach adopted in this study may provide a useful framework for future investigations, particularly when complemented by additional datasets and external validation, avoiding unsupported claims regarding the robustness of the classification results. |
| Reviewer 2 | 1. Fig 3: The flowchart uses a multi-column grid layout, but the reading direction is not indicated. There are no arrows, no numbered steps, and no visual hierarchy. The figure functions more like a scattered bullet-point list than a proper flowchart. | Thank you for this valuable suggestion. We agree that the original version of Figure 3 did not clearly convey the intended analytical workflow. The figure has been completely redesigned to improve readability and visual organization. The revised version now includes a clear sequential workflow with directional arrows, numbered stages, an improved visual hierarchy, and a more structured layout, making the progression of the analytical framework easier to follow. | Figure 3 was completely redesigned to improve clarity and readability. The revised figure includes numbered stages, directional arrows indicating the reading sequence, a clearer visual hierarchy, and a more organized layout that better represents the progression of the analytical workflow from data input to future research directions. |
| Reviewer 2 | 2. Lines 453-456, “highlighting the importance of cumulative...” Such claims are made throughout the manuscript. Findings reinforce the need to.. or highlighting the importance, but there's limited discussion on how and why the findings have these implications. | Thank you for this insightful comment. We agree that the original discussion did not sufficiently explain how the study findings support their broader implications. We have revised this section to explicitly connect the observed classification rules with our interpretation of cumulative environmental exposure. The revised text now explains that the frequent inclusion of environmental variables accumulated over 30–60-day periods in the classification rules provides the basis for emphasizing cumulative exposure, rather than isolated daily conditions, as a relevant characteristic of the environmental context associated with premature cardiovascular mortality. | The Discussion section was revised to strengthen the interpretation of the findings. Additional text was included to explain that the predominance of environmental variables accumulated over 30–60-day periods in the classification rules supports the interpretation that cumulative environmental conditions may provide more informative patterns than isolated daily fluctuations when investigating premature cardiovascular mortality. This revision provides a clearer rationale for the implications drawn from the study findings while avoiding unsupported generalizations. |
| Reviewer 2 | 3. Lines 453-456, This goes against the literature. Previous studies have shown that in such temperature ranges there is the least increase in AMI. Even in the references provided, there is either no temperature ranges provided or they refer to >35C heat waves (Chen et al.). | Thank you for this important observation. We agree that the original discussion overstated the interpretation of the identified temperature range and was not fully supported by the cited literature. Our intention was not to suggest that intermediate temperatures (22–26 °C) represent an established risk interval for AMI, but rather that this temperature range emerged as one of the environmental conditions identified by the classification model in combination with other environmental variables. We have therefore revised the discussion to avoid attributing increased AMI risk to this specific temperature interval and to clarify that these findings should be interpreted as exploratory associations identified by the classification model, rather than evidence of specific temperature-risk thresholds. | The Discussion section was revised to remove the interpretation that intermediate maximum temperatures (22–26 °C) represent an established risk range for premature AMI mortality. The revised text now clarifies that these temperatures were identified as part of multivariable classification rules and should be interpreted as exploratory environmental conditions identified by the model in combination with other variables, rather than as independent temperature thresholds associated with increased AMI risk. |
| Reviewer 2 | 4. Lines 460-462, The citations provide evidence for inverse relationship of solar radiation (either direct or indirect). Same also with this 10.1161/JAHA.119.012551. The authors state the opposite in the discussion. | Thank you for this important observation. We agree that the original discussion overstated the interpretation of the solar radiation findings and did not accurately reflect the evidence provided by the cited literature. Our intention was not to suggest that higher solar radiation is independently associated with increased AMI mortality, but rather that solar radiation emerged as one of the environmental variables composing the multivariable classification rules identified by the model. We have revised the discussion to remove this unsupported interpretation and to clarify that solar radiation should be interpreted as part of a combination of environmental conditions identified by the classification model, rather than as an independent risk factor. | The Discussion section was revised to remove the interpretation that higher solar radiation is associated with increased premature AMI mortality. The revised text now clarifies that solar radiation was identified as one component of multivariable classification rules, together with other environmental variables, and that these findings represent exploratory environmental patterns identified by the classification model rather than evidence of an independent association. |
| Reviewer 2 | 5. Lines 464-466: Repetitive statement throughout the manuscript | Thank you for this helpful observation. We agree that this section repeated concepts already discussed elsewhere in the manuscript, particularly regarding the exploratory nature of the proposed approach and the need for future validation studies. To improve conciseness and readability, we removed the repetitive statements and condensed the discussion while preserving the interpretation of the findings. | The repetitive statements in the Discussion were removed, and the affected paragraphs were condensed to improve clarity, reduce redundancy, and enhance the overall flow of the manuscript. |
| Reviewer 2 | 6. Lines 467-468: same repeating statement | Thank you for this comment. We agree that this passage reiterated concepts already presented elsewhere in the Discussion. To improve the manuscript's conciseness and readability, we revised the text by removing the redundant statements and streamlining the discussion while maintaining the scientific interpretation of the results. | The repetitive statements in this section of the Discussion were removed, and the text was condensed to improve clarity, reduce redundancy, and enhance the overall flow of the manuscript. |
| Reviewer 2 | 7. Lines 489-491: repeating the same statement again, with no evidence to back it up | Thank you for this observation. We agree that this statement repeated concepts presented elsewhere in the Discussion and was not sufficiently supported by the results. To improve clarity and avoid overinterpretation, we removed the redundant statement and revised the paragraph to focus only on interpretations directly supported by the findings | The repetitive statement was removed from the Discussion, and the paragraph was revised to retain only interpretations directly supported by the study results. |
| Reviewer 2 | Conclusion The Conclusion overclaims model utility: The Conclusion states the framework "may contribute to the development of complementary tools for monitoring population health and anticipating periods of increased cardiovascular vulnerability", yet all AMI rules have only medium confidence (59–64%) and the CVD classifier produced no valid rules at all. This framing is not warranted by the results. | Thank you for this valuable comment. We agree that the original Conclusion overstated the practical utility of the proposed framework. Our intention was not to imply that the current models are suitable for operational population health monitoring or for anticipating periods of increased cardiovascular vulnerability. We therefore revised the Conclusion to emphasize the methodological contribution of the study as an exploratory framework for identifying interpretable associations and generating hypotheses for future research. We also highlight the need for additional variables and external validation before any practical application can be considered. | The Conclusion was revised to remove statements implying direct applicability for population health monitoring or anticipation of periods of increased cardiovascular vulnerability. The revised text now emphasizes the methodological contribution of the proposed framework, its role in hypothesis generation, and the need for future studies incorporating additional variables and external validation before practical implementation. |

## Final Statement

We believe that the revisions have substantially improved the quality and clarity of the manuscript. We sincerely appreciate the Editor's and Reviewers' constructive comments and hope that the revised version is now suitable for publication.
