# Supplementary material for: Climate-sensitive patterns of premature cardiovascular mortality in a subtropical city
Source: PLoS One. 2026 Oct 28;21(10):e0357804. doi: 10.1371/journal.pone.0357804 (PMC13716314; doi:10.1371/journal.pone.0357804)
Supplement: Peer review history

# Peer review: Decision Letter 0

**DOI:** 10.1371/journal.pone.0357804.r001

**Authors:** Diego Augusto Santos Silva

9 Jun 2026

Dear Dr. Barcellos,

Thank you for submitting your manuscript to PLOS ONE. After careful consideration, we feel that it has merit but does not fully meet PLOS ONE’s publication criteria as it currently stands. Therefore, we invite you to submit a revised version of the manuscript that addresses the points raised during the review process.

Please submit your revised manuscript by Jul 24 2026 11:59PM. If you will need more time than this to complete your revisions, please reply to this message or contact the journal office at plosone@plos.org. When you're ready to submit your revision, log on to https://www.editorialmanager.com/pone/ and select the 'Submissions Needing Revision' folder to locate your manuscript file.

- A letter that responds to each point raised by the academic editor and reviewer(s). You should upload this letter as a separate file labeled 'Response to Reviewers'.
- A marked-up copy of your manuscript that highlights changes made to the original version. You should upload this as a separate file labeled 'Revised Manuscript with Track Changes'.
- An unmarked version of your revised paper without tracked changes. You should upload this as a separate file labeled 'Manuscript'.

If you would like to make changes to your financial disclosure, please include your updated statement in your cover letter. Guidelines for resubmitting your figure files are available below the reviewer comments at the end of this letter.

If applicable, we recommend that you deposit your laboratory protocols in protocols.io to enhance the reproducibility of your results. Protocols.io assigns your protocol its own identifier (DOI) so that it can be cited independently in the future. For instructions see: https://journals.plos.org/plosone/s/submission-guidelines#loc-laboratory-protocols. Additionally, PLOS ONE offers an option for publishing peer-reviewed Lab Protocol articles, which describe protocols hosted on protocols.io. Read more information on sharing protocols at https://plos.org/protocols?utm_medium=editorial-email&utm_source=authorletters&utm_campaign=protocols.

As the corresponding author, your ORCID iD is verified in the submission system and will appear in the published article. PLOS supports the use of ORCID, and we encourage all coauthors to register for an ORCID iD and use it as well. Please encourage your coauthors to verify their ORCID iD within the submission system before final acceptance, as unverified ORCID iDs will not appear in the published article. *Only*  the individual author can complete the verification step; PLOS staff *cannot*  verify ORCID iDs on behalf of authors.

We look forward to receiving your revised manuscript.

Kind regards,

Diego Augusto Santos Silva, Ph.D.

Academic Editor

PLOS One

Journal Requirements:

When submitting your revision, we need you to address these additional requirements.

1. Please ensure that your manuscript meets PLOS ONE's style requirements, including those for file naming. The PLOS ONE style templates can be found at

https://journals.plos.org/plosone/s/file?id=wjVg/PLOSOne_formatting_sample_main_body.pdf and

https://journals.plos.org/plosone/s/file?id=ba62/PLOSOne_formatting_sample_title_authors_affiliations.pdf

2. Please note that PLOS One has specific guidelines on code sharing for submissions in which author-generated code underpins the findings in the manuscript. In these cases, we expect all author-generated code to be made available without restrictions upon publication of the work. Please review our guidelines at https://journals.plos.org/plosone/s/materials-and-software-sharing#loc-sharing-code and ensure that your code is shared in a way that follows best practice and facilitates reproducibility and reuse.

3. In the online submission form, you indicated that your data is available only on request from a third party. Please note that your Data Availability Statement is currently missing contact details for the third party, such as an email address or a link to where data requests can be made. Please update your statement with the missing information.

4. Please upload a new copy of Figures 1 - 3 as the detail is not clear. Please follow the link for more information:  https://journals.plos.org/plosone/s/figures

5. Please include captions for your Supporting Information files at the end of your manuscript, and update any in-text citations to match accordingly. Please see our Supporting Information guidelines for more information: http://journals.plos.org/plosone/s/supporting-information.

6. If the reviewer comments include a recommendation to cite specific previously published works, please review and evaluate these publications to determine whether they are relevant and should be cited. There is no requirement to cite these works unless the editor has indicated otherwise.

Additional Editor Comments:

Dear Authors,

Two external reviewers have evaluated your article and recommended changes to the manuscript. Please see the reviewers' comments below and make the necessary corrections.

Reviewer 1

Dear authors, I have reviewed your manuscript and find it to be an excellent work; however, I have observed that some minor adjustments should be made:

1. Innovation in Methodology

The great success of the study lies in replacing traditional linear statistics with Data Mining (Artificial Intelligence). Since climate and human health are related in a complex and non-linear way, the use of algorithms such as K-means and CBA proved to be the right choice for finding patterns that would be missed in common analyses.

2. Importance of Lags (Time Delays)

The creation of 90 lag variables (cumulative effects of 2 to 60 days) was a crucial methodological success. The impact of climate on the human body is rarely immediate; the study proves that cardiovascular stress is a reflection of meteorological conditions that linger or vary over days and weeks.

3. Dynamics of Results (Climate and the Heart)

The study managed to separate the "atmospheric behavior" of two serious conditions:

Ischemic Heart Disease (IHD): Showed to be linked to chronic stress from prolonged cold and high humidity.

Acute Myocardial Infarction (AMI): Showed to be more acute, associated with lower atmospheric pressure (common in abrupt weather changes and cold fronts), which can destabilize the circulatory system.

4. Limitations for Adjustment

Incomplete data: The final section on AMI (which had the best accuracy, 70.25%) was cut from the presented text and needs to be detailed.

Missing variables: The model would greatly benefit from including air pollution, which tends to increase in winter along with heart disease.

Practical Conclusion

The article is excellent for generating hypotheses and has immediate real-world application. These weather patterns can be used by governments to create Early Warning Systems, allowing hospitals and vulnerable groups to prepare days before a critical weather event.

Reviewer 2

The manuscript titled “Climate-sensitive patterns of premature cardiovascular mortality in a

subtropical city” investigates associations between daily climatic conditions and premature mortality from CVD, IHD and AMI in Curitiba, Brazil, over the period spanning 2010-2019. The authors present an exploratory, data-driven framework which combines multivariate analysis, k-means and hierarchical clustering, and classification-based association rule mining. Even though the research is interesting and is based on a substantial dataset (spans 10 years), the manuscript has several significant methodological weaknesses that must be addressed.

Introduction:

1. The first four paragraphs of the introduction are short, repetitive and light on references (only 1 is used). I suggest to combine them in a single concise paragraph with more substantial information.

2. Lines 88-89: How has climate change affected urban temperatures specifically?

3. The paragraph that spans lines 94-103 makes significant statements but only refers to the same paper again (ref. 1).

4. Lines 106-108: Be more specific about past research. What does the literature state about this association? which previous studies, what were the outcomes of these studies?

5. Line 110, Recent studies reinforce... Which recent studies? The same reference is used quite a lot in the introduction ([1]).

6. Sentence in line 113: Need to expand this. Which previous studies, which data mining techniques etc..?

7. Line 126, “Current findings indicate...”, need to summarise current findings with appropriate citations.

8. Line 127, “New approaches...” which new approaches? what were the findings?

9. Line 135, “..allows for an understanding..”, remove “an”

Methods

1. Paragraphs spanning lines 139-153 belong in the introduction.

2. Line 167, SMS was not defined before

3. Line 180. Only a single station is used. While this is not uncommon in similar studies, the authors make no effort to describe the relevancy of this station to the rest of the city. Where is it located with respect with the city etc. The authors themselves cite evidence that heat islands were documented in Curitiba in 2019, with temperatures in the urban core exceeding 29°C and temperatures in vegetated areas up to 10°C lower. This documented within-city thermal heterogeneity means that the single-station exposure values may systematically misclassify the actual temperature experienced by residents in different neighbourhoods. This is particularly consequential for AMI, where the identified temperature range of 22–26°C spans only four degrees, a range fully encompassed by the within-city variability the authors acknowledge. The authors should also consider whether multi-station data (additional SIMEPAR stations in Curitiba's metropolitan area) or gridded reanalysis products (e.g., ERA5) could be used to test the sensitivity of the classification rules to the choice of exposure data.

4. What is the justification for the use of these variables (Table 1).

5. The paragraph spanning lines 194-197 is redundant, remove it.

6. What is the justification for using lag times up to 60 days?

7. Lines 206-208, how were these calculated?

8. Discretization is an important and common preprocessing step, but it is not universally necessary for all data mining tasks. Many algorithms (e.g., linear regression, support vector machines, neural networks) work directly with continuous variables without requiring discretization.

9. In general this section contains repetitive statements and not nearly enough details to reproduce the experiment. (Data Preparation section).

CBA is quite dated. why use this methods and not something more contemporary?

10. e.g. random forest, xgboost. These could be wrapped in a SHAP-model for further explainability etc..

11. Line 254, why different thresholds for CVD deaths? The authors do not discuss this.

12. Line 257, repetitive statement, remove.

13. Lines 274-275: why different pruning control parameters?

14. For the model evaluation, no held-out portion, no cross-validation? How can these be trusted that it's not overfitting?

Results

1. The authors should explicitly state:

- Why the 90 derived/lagged variables were excluded from the correlation matrix

- Whether this selective inclusion could have biased the variable relationships presented

2. Fig 1: The authors acknowledge the high inter-variable correlations (r > 0.7) in a single sentence, dismissing them as physically expected, but they provide no methodological response to this finding. They do not apply dimensionality reduction, variable selection, or any correction before feeding all variables into the clustering and classification models.

Also for the modelling part K-means uses Euclidean distances, which are distorted by redundant variables. Highly correlated variables effectively get double- or triple-weighted in the distance calculation. For instance, including avg_pressure, min_pressure, and max_pressure (all r > 0.97 ) means atmospheric pressure dominates cluster formation disproportionately.

For the regression-based modelling part Multicollinearity inflates standard errors, destabilizes coefficient estimates, and reduces statistical power, making it difficult to isolate the independent contribution of each variable. For example, if avg_temp, min_temp, and max_temp (all r > 0.89 with each other ) are all included in a regression, their individual coefficients become unreliable. For the CBA classification model too, feeding highly correlated discretised variables (e.g., avg_pressure, min_pressure, max_pressure all in terciles) means the algorithm is effectively sampling the same underlying signal three times when generating rules, inflating apparent rule support.

3. why all 104 variables are used here and not in the previous experiment? (line 305)

4. The sentences in lines 320-321 is repetitive and redundant, remove.

5. Fig 2: Caption states 104 whereas diagram title 107. which one is correct?

6. Fig 2: the x-axis is not readable

7. Line 328, “It was identified..” how was this identified? Why not use standard methods such as the elbow method or the silhouette scores method?

8. Table 2: Cluster 2 is oddly heterogeneous, discuss. is the accumulated precipitation over the past 60 days a really relevant feature?

9. Lines 348-349, “The model generated...” then why were these included? Have you tried running the model with a simpler rule (like the IHD and AMI cases)?

10. In table 3 the CVD rules are all below the thresholds set by the authors. Why include them?

11. Line 363, “demonstrating its robustness..” How is this demonstrating its robustness?

12. Line 375, show should be shows

13. Line 386 “Nonetheless, recognizing this limitation...” I disagree with this statement. The results presented for the classification model do not seem to be robust, so how can they be used in comparisons in future studies?

Discussion

1. Fig 3: The flowchart uses a multi-column grid layout, but the reading direction is not indicated. There are no arrows, no numbered steps, and no visual hierarchy. The figure functions more like a scattered bullet-point list than a proper flowchart.

2. Lines 453-456, “highlighting the importance of cumulative...” Such claims are made throughout the manuscript. Findings reinforce the need to.. or highlighting the importance, but there's limited discussion on how and why the findings have these implications.

3. Lines 453-456, This goes against the literature. Previous studies have shown that in such temperature ranges there is the least increase in AMI. Even in the references provided, there is either no temperature ranges provided or they refer to >35C heat waves (Chen et al.).

4. Lines 460-462, The citations provide evidence for inverse relationship of solar radiation (either direct or indirect). Same also with this 10.1161/JAHA.119.012551. The authors state the opposite in the discussion.

5. Lines 464-466: Repetitive statement throughout the manuscript

6. Lines 467-468: same repeating statement

7. Lines 489-491: repeating the same statement again, with no evidence to back it up

Conclusion

The Conclusion overclaims model utility: The Conclusion states the framework "may contribute to the development of complementary tools for monitoring population health and anticipating periods of increased cardiovascular vulnerability", yet all AMI rules have only medium confidence (59–64%) and the CVD classifier produced no valid rules at all. This framing is not warranted by the results.

[Note: HTML markup is below. Please do not edit.]

Reviewers' comments:

Reviewer's Responses to Questions

**Comments to the Author**

1. Is the manuscript technically sound, and do the data support the conclusions?

Reviewer #1: Yes

Reviewer #2: No

**********

2. Has the statistical analysis been performed appropriately and rigorously?

Reviewer #1: Yes

Reviewer #2: No

**********

3. Have the authors made all data underlying the findings in their manuscript fully available?

The PLOS Data policy

Reviewer #1: Yes

Reviewer #2: No

**********

4. Is the manuscript presented in an intelligible fashion and written in standard English?

Reviewer #1: Yes

Reviewer #2: Yes

**********

Reviewer #1: Dear authors, I have reviewed your manuscript and find it to be an excellent work; however, I have observed that some minor adjustments should be made:

1. Innovation in Methodology

The great success of the study lies in replacing traditional linear statistics with Data Mining (Artificial Intelligence). Since climate and human health are related in a complex and non-linear way, the use of algorithms such as K-means and CBA proved to be the right choice for finding patterns that would be missed in common analyses.

2. Importance of Lags (Time Delays)

The creation of 90 lag variables (cumulative effects of 2 to 60 days) was a crucial methodological success. The impact of climate on the human body is rarely immediate; the study proves that cardiovascular stress is a reflection of meteorological conditions that linger or vary over days and weeks.

3. Dynamics of Results (Climate and the Heart)

The study managed to separate the "atmospheric behavior" of two serious conditions:

Ischemic Heart Disease (IHD): Showed to be linked to chronic stress from prolonged cold and high humidity.

Acute Myocardial Infarction (AMI): Showed to be more acute, associated with lower atmospheric pressure (common in abrupt weather changes and cold fronts), which can destabilize the circulatory system.

4. Limitations for Adjustment

Incomplete data: The final section on AMI (which had the best accuracy, 70.25%) was cut from the presented text and needs to be detailed.

Missing variables: The model would greatly benefit from including air pollution, which tends to increase in winter along with heart disease.

Practical Conclusion

The article is excellent for generating hypotheses and has immediate real-world application. These weather patterns can be used by governments to create Early Warning Systems, allowing hospitals and vulnerable groups to prepare days before a critical weather event.

Reviewer #2: The manuscript titled “Climate-sensitive patterns of premature cardiovascular mortality in a

subtropical city” investigates associations between daily climatic conditions and premature mortality from CVD, IHD and AMI in Curitiba, Brazil, over the period spanning 2010-2019. The authors present an exploratory, data-driven framework which combines multivariate analysis, k-means and hierarchical clustering, and classification-based association rule mining. Even though the research is interesting and is based on a substantial dataset (spans 10 years), the manuscript has several significant methodological weaknesses that must be addressed.

Introduction:

1. The first four paragraphs of the introduction are short, repetitive and light on references (only 1 is used). I suggest to combine them in a single concise paragraph with more substantial information.

2. Lines 88-89: How has climate change affected urban temperatures specifically?

3. The paragraph that spans lines 94-103 makes significant statements but only refers to the same paper again (ref. 1).

4. Lines 106-108: Be more specific about past research. What does the literature state about this association? which previous studies, what were the outcomes of these studies?

5. Line 110, Recent studies reinforce... Which recent studies? The same reference is used quite a lot in the introduction ([1]).

6. Sentence in line 113: Need to expand this. Which previous studies, which data mining techniques etc..?

7. Line 126, “Current findings indicate...”, need to summarise current findings with appropriate citations.

8. Line 127, “New approaches...” which new approaches? what were the findings?

9. Line 135, “..allows for an understanding..”, remove “an”

Methods

1. Paragraphs spanning lines 139-153 belong in the introduction.

2. Line 167, SMS was not defined before

3. Line 180. Only a single station is used. While this is not uncommon in similar studies, the authors make no effort to describe the relevancy of this station to the rest of the city. Where is it located with respect with the city etc. The authors themselves cite evidence that heat islands were documented in Curitiba in 2019, with temperatures in the urban core exceeding 29°C and temperatures in vegetated areas up to 10°C lower. This documented within-city thermal heterogeneity means that the single-station exposure values may systematically misclassify the actual temperature experienced by residents in different neighbourhoods. This is particularly consequential for AMI, where the identified temperature range of 22–26°C spans only four degrees, a range fully encompassed by the within-city variability the authors acknowledge. The authors should also consider whether multi-station data (additional SIMEPAR stations in Curitiba's metropolitan area) or gridded reanalysis products (e.g., ERA5) could be used to test the sensitivity of the classification rules to the choice of exposure data.

4. What is the justification for the use of these variables (Table 1).

5. The paragraph spanning lines 194-197 is redundant, remove it.

6. What is the justification for using lag times up to 60 days?

7. Lines 206-208, how were these calculated?

8. Discretization is an important and common preprocessing step, but it is not universally necessary for all data mining tasks. Many algorithms (e.g., linear regression, support vector machines, neural networks) work directly with continuous variables without requiring discretization.

9. In general this section contains repetitive statements and not nearly enough details to reproduce the experiment. (Data Preparation section).

CBA is quite dated. why use this methods and not something more contemporary?

10. e.g. random forest, xgboost. These could be wrapped in a SHAP-model for further explainability etc..

11. Line 254, why different thresholds for CVD deaths? The authors do not discuss this.

12. Line 257, repetitive statement, remove.

13. Lines 274-275: why different pruning control parameters?

14. For the model evaluation, no held-out portion, no cross-validation? How can these be trusted that it's not overfitting?

Results

1. The authors should explicitly state:

- Why the 90 derived/lagged variables were excluded from the correlation matrix

- Whether this selective inclusion could have biased the variable relationships presented

2. Fig 1: The authors acknowledge the high inter-variable correlations (r > 0.7) in a single sentence, dismissing them as physically expected, but they provide no methodological response to this finding. They do not apply dimensionality reduction, variable selection, or any correction before feeding all variables into the clustering and classification models.

Also for the modelling part K-means uses Euclidean distances, which are distorted by redundant variables. Highly correlated variables effectively get double- or triple-weighted in the distance calculation. For instance, including avg_pressure, min_pressure, and max_pressure (all r > 0.97 ) means atmospheric pressure dominates cluster formation disproportionately.

For the regression-based modelling part Multicollinearity inflates standard errors, destabilizes coefficient estimates, and reduces statistical power, making it difficult to isolate the independent contribution of each variable. For example, if avg_temp, min_temp, and max_temp (all r > 0.89 with each other ) are all included in a regression, their individual coefficients become unreliable. For the CBA classification model too, feeding highly correlated discretised variables (e.g., avg_pressure, min_pressure, max_pressure all in terciles) means the algorithm is effectively sampling the same underlying signal three times when generating rules, inflating apparent rule support.

3. why all 104 variables are used here and not in the previous experiment? (line 305)

4. The sentences in lines 320-321 is repetitive and redundant, remove.

5. Fig 2: Caption states 104 whereas diagram title 107. which one is correct?

6. Fig 2: the x-axis is not readable

7. Line 328, “It was identified..” how was this identified? Why not use standard methods such as the elbow method or the silhouette scores method?

8. Table 2: Cluster 2 is oddly heterogeneous, discuss. is the accumulated precipitation over the past 60 days a really relevant feature?

9. Lines 348-349, “The model generated...” then why were these included? Have you tried running the model with a simpler rule (like the IHD and AMI cases)?

10. In table 3 the CVD rules are all below the thresholds set by the authors. Why include them?

11. Line 363, “demonstrating its robustness..” How is this demonstrating its robustness?

12. Line 375, show should be shows

13. Line 386 “Nonetheless, recognizing this limitation...” I disagree with this statement. The results presented for the classification model do not seem to be robust, so how can they be used in comparisons in future studies?

Discussion

1. Fig 3: The flowchart uses a multi-column grid layout, but the reading direction is not indicated. There are no arrows, no numbered steps, and no visual hierarchy. The figure functions more like a scattered bullet-point list than a proper flowchart.

2. Lines 453-456, “highlighting the importance of cumulative...” Such claims are made throughout the manuscript. Findings reinforce the need to.. or highlighting the importance, but there's limited discussion on how and why the findings have these implications.

3. Lines 453-456, This goes against the literature. Previous studies have shown that in such temperature ranges there is the least increase in AMI. Even in the references provided, there is either no temperature ranges provided or they refer to >35C heat waves (Chen et al.).

4. Lines 460-462, The citations provide evidence for inverse relationship of solar radiation (either direct or indirect). Same also with this 10.1161/JAHA.119.012551. The authors state the opposite in the discussion.

5. Lines 464-466: Repetitive statement throughout the manuscript

6. Lines 467-468: same repeating statement

7. Lines 489-491: repeating the same statement again, with no evidence to back it up

Conclusion

The Conclusion overclaims model utility: The Conclusion states the framework "may contribute to the development of complementary tools for monitoring population health and anticipating periods of increased cardiovascular vulnerability", yet all AMI rules have only medium confidence (59–64%) and the CVD classifier produced no valid rules at all. This framing is not warranted by the results.

**********

what does this mean?). If published, this will include your full peer review and any attached files.

If you choose “no”, your identity will remain anonymous but your review may still be made public.

**Do you want your identity to be public for this peer review?**  For information about this choice, including consent withdrawal, please see our Privacy Policy

Reviewer #1: **Yes:**  GIVANILDO DE GOIS

Reviewer #2: No

**********

[NOTE: If reviewer comments were submitted as an attachment file, they will be attached to this email and accessible via the submission site. Please log into your account, locate the manuscript record, and check for the action link "View Attachments". If this link does not appear, there are no attachment files.]

To ensure your figures meet our technical requirements, please review our figure guidelines: https://journals.plos.org/plosone/s/figures

You may also use PLOS’s free figure tool, NAAS, to help you prepare publication quality figures: https://journals.plos.org/plosone/s/figures#loc-tools-for-figure-preparation.

NAAS will assess whether your figures meet our technical requirements by comparing each figure against our figure specifications.

# Author comment: Author response to Decision Letter 1

**DOI:** 10.1371/journal.pone.0357804.r002

30 Jul 2026

A detailed point-by-point response to all reviewer and editor comments has been provided in the attached "Response to Reviewers" document. The revised manuscript has been updated accordingly.

Attachment Submitted filename: Response_to_Reviewers_Professional.docx

# Editor report: Decision Letter 1

**DOI:** 10.1371/journal.pone.0357804.r003

**Authors:** Diego Augusto Santos Silva

23 Aug 2026

Climate-sensitive patterns of premature cardiovascular mortality in a subtropical city

PONE-D-26-00863R1

Dear Dr. Barcellos,

We’re pleased to inform you that your manuscript has been judged scientifically suitable for publication and will be formally accepted for publication once it meets all outstanding technical requirements.

Within one week, you’ll receive an e-mail detailing the required amendments. When these have been addressed, you’ll receive a formal acceptance letter and your manuscript will be scheduled for publication.

An invoice will be generated when your article is formally accepted. Please note, if your institution has a publishing partnership with PLOS and your article meets the relevant criteria, all or part of your publication costs will be covered. Please make sure your user information is up-to-date by logging into Editorial Manager at Editorial Manager® and clicking the ‘Update My Information' link at the top of the page. For questions related to billing, please contact billing support.

If your institution or institutions have a press office, please notify them about your upcoming paper to help maximize its impact. If they’ll be preparing press materials, please inform our press team as soon as possible -- no later than 48 hours after receiving the formal acceptance. Your manuscript will remain under strict press embargo until 2 pm Eastern Time on the date of publication. For more information, please contact onepress@plos.org.

Kind regards,

Diego Augusto Santos Silva, Ph.D.

Academic Editor

PLOS One

Additional Editor Comments (optional):

The authors revised the manuscript in accordance with the reviewers' recommendations. Indeed, the authors made a significant effort to improve the work and address the reviewers' requests, and, in my opinion, they did so satisfactorily. I consider the manuscript suitable for publication.

Reviewers' comments:

# Editor report: Acceptance letter

**DOI:** 10.1371/journal.pone.0357804.r004

**Authors:** Diego Augusto Santos Silva

PONE-D-26-00863R1

PLOS One

Dear Dr. Barcellos,

I'm pleased to inform you that your manuscript has been deemed suitable for publication in PLOS One. Congratulations! Your manuscript is now being handed over to our production team.

At this stage, our production department will prepare your paper for publication. This includes ensuring the following:

* All references, tables, and figures are properly cited

* All relevant supporting information is included in the manuscript submission,

* There are no issues that prevent the paper from being properly typeset

You will receive further instructions from the production team, including instructions on how to review your proof when it is ready. Please keep in mind that we are working through a large volume of accepted articles, so please give us a few days to review your paper and let you know the next and final steps.

Lastly, if your institution or institutions have a press office, please let them know about your upcoming paper now to help maximize its impact. If they'll be preparing press materials, please inform our press team within the next 48 hours. Your manuscript will remain under strict press embargo until 2 pm Eastern Time on the date of publication. For more information, please contact onepress@plos.org.

You will receive an invoice from PLOS for your publication fee after your manuscript has reached the completed accept phase. If you receive an email requesting payment before acceptance or for any other service, this may be a phishing scheme. Learn how to identify phishing emails and protect your accounts at https://explore.plos.org/phishing.

If we can help with anything else, please email us at customercare@plos.org.

Thank you for submitting your work to PLOS One and supporting open access.

Kind regards,

PLOS One Editorial Office Staff

on behalf of

Dr. Diego Augusto Santos Silva

Academic Editor

PLOS One
